# The nicotinamide phosphoribosyltransferase inhibitor FK866 restricts influenza A virus replication by perturbing viral polymerase activity

**DOI:** 10.1128/jvi.00591-26

**Published:** 2026-06-15

**Authors:** Changjie Lv, Shuang Wang, Mingyue Song, Jianmei Wu, Wanxin Wei, Guijie Guo

**Affiliations:** 1 Key Laboratory of Animal Pathogen Infection and Immunology of Fujian Province, College of Animal Sciences, Fujian Agriculture and Forestry University 12449, Fuzhou, China; 2 Joint Laboratory of Animal Pathogen Prevention and Control of Fujian-Nepal, College of Animal Sciences, Fujian Agriculture and Forestry University, Fuzhou, China; 3 Key Laboratory of Fujian-Taiwan Animal Pathogen Biology, College of Animal Sciences, Fujian Agriculture and Forestry University, Fuzhou, China; 4 Engineering Research Center for Animal Breeding and Sustainable Production, College of Animal Sciences, Fujian Agriculture and Forestry University, Fuzhou, China; University of Kentucky College of Medicine, Lexington, Kentucky, USA

**Keywords:** influenza A virus, NAMPT, FK866, PB1, virus replication

## Abstract

Nicotinamide phosphoribosyltransferase (NAMPT), a key rate-limiting enzyme in NAD^+^ synthesis, plays important roles in various physiological and pathological processes. However, the function and underlying mechanisms of NAMPT in influenza A virus (IAV) infection and pathogenesis remain ambiguous. Here, *in vitro* studies showed that NAMPT had profound effects on the replication of IAV. Treatment with the NAMPT inhibitor FK866 or disruption of NAMPT expression markedly attenuated the replication of various IAV subtypes, including H1N1, H3N2, and H9N2. Additionally, FK866-treated mice exhibited significant resistance to the IAV infection, as evidenced by a lower degree of tissue injury, slower body weight loss, and better survival than untreated animals challenged with IAV. Mechanistically, NAMPT interacted with viral polymerase basic protein 1 (PB1) and promoted the association of PB1 with polymerase acidic protein (PA) and polymerase basic protein 2 (PB2). Inhibition or depletion of NAMPT restrained the activity of viral RNA-dependent RNA polymerase (RdRp), thereby repressing IAV transcription and replication. Furthermore, we identified arginine 203 of PB1 as a critical residue for its interaction with NAMPT. The mutation (arginine 203 to proline, R203P) of PB1 hindered the association of PB1 with NAMPT, PA, and PB2, thereby impairing the RdRp activity and limiting IAV transcription and replication. Collectively, these findings uncover a critical role of NAMPT in regulating IAV replication and characterize the antiviral property of the NAMPT inhibitor FK866 against IAVs, providing insights for the development of novel anti-influenza strategies.

**IMPORTANCE:**

Influenza A virus (IAV) causes acute respiratory diseases in humans and animals and poses a great threat to public health, highlighting the urgent need for more effective antiviral treatments. Here, we found that the nicotinamide phosphoribosyltransferase (NAMPT) inhibitor FK866 significantly suppressed the replication of IAV *in vitro* and *in vivo*. Of note, FK866 markedly attenuated the replication of different IAV subtypes, including H1N1, H3N2, and H9N2, suggesting FK866 as a potential broad-spectrum antiviral against IAVs. Mechanistically, NAMPT interacted with viral polymerase basic protein 1 (PB1) and enhanced the association of PB1 with PA and PB2. FK866 could interfere with the viral polymerase activity, thereby limiting the synthesis of IAV viral RNA, complementary RNA, and messenger RNA. These findings reveal that the NAMPT inhibitor FK866 restricts IAV replication by perturbing viral polymerase activity and provide insights into the development of potential antivirals against IAV infection.

## INTRODUCTION

Influenza (flu), caused by influenza viruses of the *Orthomyxoviridae* family, is a globally prevalent respiratory disease and poses a great threat to the health of humans and animals (1, 2). The four influenza virus types (A, B, C, and D) are classified based on nucleoprotein (NP) and matrix protein 1 antigenic variation, epidemiological characteristics, host range, and disease severity (3, 4). Notably, influenza A viruses (IAVs) possess unique pandemic potential due to high-frequency variations (5–7). Several disastrous IAV pandemics have resulted in millions of deaths, including the Spanish influenza pandemic, with an estimated 20–50 million deaths caused by H1N1 in 1918–1919, Asian flu caused by H2N2 subtype in 1957–1958, and Hong Kong influenza caused by H3N2 subtype in 1968–1969 (8–10). In 1997, H5N1 avian influenza spread from chickens to humans and broke out in Hong Kong, followed by its spread to Asia and other continents, causing high mortality rates and economic loss (11, 12). The pandemic H1N1-2009 emerged on 26 April 2009 as a public health emergency that led to high case-fatality rates (13, 14). These historical precedents underscore the critical need for antiviral strategies preventing IAV transmission.

The RNA-dependent RNA polymerase (RdRp) of IAV consists of polymerase basic protein 1 (PB1), polymerase basic protein 2 (PB2), and polymerase acidic protein (PA). IAV viral RNA (vRNA) segments are associated with the RdRp subunits (PB2, PB1, and PA) and encapsidated by NP to form the viral ribonucleoprotein (vRNP) complex (15, 16). The transcription and replication of the IAV genome are carried out by the vRNP complex, which could hijack and manipulate host transcriptional systems or other host cellular machinery to synthesize vRNA, complementary RNA (cRNA), and messenger RNA (mRNA) (17, 18). In turn, these essential host-virus interactions may provide promising targets for novel antiviral strategies. Numerous host proteins have been reported to regulate the migration and function of the IAV vRNP complex (19–22). However, detailed mechanisms underlying the regulation of the vRNP activity are not fully understood and deserve further investigation.

Nicotinamide phosphoribosyltransferase (NAMPT) is a rate-limiting enzyme of nicotinamide adenine dinucleotide (NAD^+^) synthesis and plays important roles in various pathological processes, including cancers and viral infections (23–28). FK866, a highly selective NAMPT inhibitor, accelerates the death of hepatitis B virus (HBV)-induced liver cancer cells, highlighting the potential of NAMPT as a therapeutic candidate for HBV-associated liver cancer (29, 30). Additionally, FK866 also suppresses colorectal cancer proliferation (31). Moreover, autophagy plays a vital role in sepsis-induced acute lung injury (ALI) (32), and FK866 could inhibit the JNK signaling pathway, thereby mitigating ALI via JNK-dependent autophagy (33). These observations suggest targeting NAMPT as a promising strategy to eliminate malignant cells or limit viral infections. However, the function of NAMPT in IAV infection and the potential of targeting NAMPT to restrict IAV infection remain largely unexplored.

In this study, we found that the NAMPT inhibitor FK866 significantly inhibited the replication of various subtypes of IAVs, including H1N1, H3N2, and H9N2. Mechanistically, FK866 interfered with the viral polymerase activity, thereby repressing the synthesis of IAV vRNA, cRNA, and mRNA. Additionally, oral administration of FK866 rendered mice more resistant to IAV infection, as evidenced by the improved survival rate, reduced pathological tissue damage, and decreased viral loads in mice following IAV infection.

## MATERIALS AND METHODS

### Cells and viruses

A549 and MDCK cells were cultured in Dulbecco’s Modified Eagle Medium culture medium (Gibco, Waltham, MA, USA) supplemented with 10% fetal bovine serum (Gibco), 100 μg/mL streptomycin, and 100 IU/mL penicillin at 37°C in a 5% CO_2_ atmosphere. The IAVs H1N1 A/PR/8/34 (PR8), H1N1 A/WSN/1933 (WSN), H9N2 A/chicken/Fujian/MQ01/2015, H3N2 A/swine/Shandong/HZNL/2023, H1N1 (CT F4), as well as the Pseudorabies virus (PRV) strain PRV (SDFB), were stored at −80°C until use.

### Cell counting Kit-8 assay

Cell viability assays were performed according to the manufacturer’s instructions (catalog no. GK10001; GlpBio, Montclair, USA). In brief, A549 cells were seeded in a 96-well plate and treated with FK866 or vehicle of different concentrations for 24 h. Then, 10 μL CCK-8 reagent was added to each well, followed by incubation at 37°C for 30 min. The absorbance was measured at 450 nm.

### Antibodies and reagents

Rabbit antibodies against IAV NP, PB2, PA, and PB1 were purchased from Gene Tex (catalog no. GTX14213, 1:1,000; GTX125926, 1:5,000; GTX118991, 1:1,000; GTX637313, 1:1,000; Southern California, USA). The mouse anti-GAPDH antibody was purchased from Proteintech (catalog no. 60004-1-Ig; 1:10,000; Wuhan, China). The rabbit anti-HA tag antibody was purchased from Proteintech (catalog no. 81290-1-RR; 1:10,000). The rabbit anti-Flag tag antibody was purchased from GenScript (catalog no. A00170; 1:1,000; Nanjing, China). The mouse-derived antibody against PRV gB was stored in our laboratory and used at a 1:5,000 dilution. The rabbit anti-NAMPT monoclonal antibody was purchased from Cell Signaling Technology (catalog no. 61122S; 1:5,000; Massachusetts, USA). FK866 was purchased from GlpBio.

### TCID_50_ assay

IAV titers were measured using TCID_50_ assays. For tissue samples, lungs (0.2 g per mouse) were homogenized in 600 µL PBS. Following centrifugation, the clarified supernatants were harvested. In addition, cell culture supernatants were collected from each treatment group. MDCK cells were seeded in a 96-well plate. One hundred microliters (100 µL) of 10-fold serial dilutions of IAV was added to the cells, with eight replicates per dilution, and incubated for 1 h. Subsequently, the inoculum was removed, and the cells were washed three times with PBS. Finally, the culture medium was replaced with fresh medium containing 2% FBS. Then, the 20 μL cell culture supernatant was mixed with 1% chicken red blood cells with equal volume in V-bottom plates after 72 hpi. The mixture was incubated at 37°C for 30 min, and the results were calculated using the Reed-Muench method.

### Western blotting

The proteins were separated by the SDS-PAGE kit from Vazyme (Nanjing, China). After SDS-PAGE, proteins were transferred to nitrocellulose membranes (Waukesha, USA). Membranes were blocked with 5% skim milk in Tris-buffered saline with Tween 20 (TBST) at 37°C for 1 h. Then, membranes were incubated with corresponding primary antibodies overnight at 4°C, followed by washing with TBST five times. The membranes were incubated with HRP-conjugated secondary antibodies at 37°C for 1 h and washed with TBST five times. The ECL substrate was applied, and signals were acquired using the Tanon-5200 Imaging System (Shanghai, China).

### RNA extraction, RT-PCR, and quantitative real-time PCR

The mRNA levels were quantified using qPCR. The lung samples (0.2 g per mouse) were homogenized in 600 µL PBS. After centrifugation, the clarified supernatants were collected. Cells from each treatment group, as indicated, were harvested. The total RNA was extracted using the total RNA isolation kit from Magen (Guangzhou, China), according to the manufacturer’s instructions. RNA samples were reverse-transcribed into cDNA using the reverse transcription kit (Vazyme) following the manufacturer’s protocol. The cDNA served as templates for qPCR using a QuantStudio 1 Plus detection system (ABI, Waltham, MA, USA). GAPDH was used as an endogenous control for normalization. The expression of each target gene was calculated using the 2^−ΔΔCT^ method. The qPCR primer sequences are listed in Table S1.

### NAMPT activity assay

NAMPT activity assays were performed using the Colorimetric Assay Kit (version 2, catalog no. CY-1251V2; CycLex (MBL), Japan). A549 cells were infected with H1N1 (PR8), H3N2, or H9N2 for 1 h. Then, the viral inoculum was removed, and the cells were washed three times with PBS. Subsequently, medium containing either FK866 or vehicle was added and incubated for 24 h. Finally, the samples were collected and processed for detection according to the manufacturer’s instructions.

### Plasmids and short hairpin RNA

The pCAGGS-Flag plasmids encoding the PA, PB1, PB2, and NP proteins of the H1N1 (PR8) virus, as well as the pHW2000 plasmids for PA, PB2, NP, and PB1, were generated previously in our laboratory. NAMPT cDNA was constructed into the pCAGGS-HA vector. The PB1 mutant (R203P) or the PB2 mutant (E361A) was cloned into the pCAGGS-Flag vector. The NAMPT shRNA was cloned into the pLKO.1 vector. The pPol I-NP-vRNA and pPol I-NA-vRNA plasmids were kindly provided by Prof. Qiyun Zhu (Lanzhou Veterinary Research Institute, Chinese Academy of Agricultural Sciences, China). The sequences of PCR primers and short hairpin RNA are listed in Table S1.

### Immunoprecipitation assay

HEK293T cells were co-transfected with the indicated plasmids using the Lipo8000 transfection reagent according to the manufacturer’s instructions (catalog no. C0533; Beyotime, Shanghai, China). At 24 h post-transfection, the cells were lysed on ice with immunoprecipitation (IP) lysis buffer containing a protease inhibitor cocktail (catalog no. IKM1140; Solarbio, Beijing, China). The clarified lysates were then subjected to immunoprecipitation using anti-Flag or anti-HA magnetic beads (catalog no. HY-K0207, HY-K0201; MCE, Shanghai, China). Finally, the immunoprecipitated proteins and the corresponding input lysates were subjected to Western blotting analysis.

### Molecular docking model

Protein sequences were obtained from UniProt (34). The protein sequences used for homology modeling were retrieved from the UniProt Knowledgebase under the following accession numbers: P03433, influenza A virus (strain A/Puerto Rico/8/1934 H1N1) PA (https://www.uniprot.org/uniprotkb/P03433/entry); P03431, influenza A virus (strain A/Puerto Rico/8/1934 H1N1) PB1 (https://www.uniprot.org/uniprotkb/P03431/entry); and P43490, human NAMPT (https://www.uniprot.org/uniprotkb/P43490/entry). Structures were homology-modeled with ProteinX and energy-minimized using Rosetta Relax. PyMOL was used to prepare the resulting models (removing water and adding hydrogens) for subsequent computations. Initial rigid-body docking was carried out with HDOCK, followed by RosettaDock for flexible refinement starting from the HDOCK poses. Models were selected based on the Rosetta energy score. Protein-protein interactions were mapped with LigPlot, and the overall binding energy was calculated using Rosetta’s Interface_analyzer module. Final complexes were visualized in PyMOL.

### Molecular dynamics and virtual mutagenesis

The docked NAMPT-PB1 protein complex served as the initial structure for molecular dynamics simulations performed with Gromacs 2023.3. Prior to simulation, the system underwent energy minimization. Following minimization, a 100 ps NVT simulation was conducted to gradually heat the system from 0 K to 310.15 K at constant volume, allowing for further equilibration of solvent distribution. This was followed by a 100 ps NPT simulation using the Berendsen barostat for pressure equilibration. During the production molecular dynamics simulations, all bonds involving hydrogen were constrained using the LINCS algorithm with an integration time step of 2 fs. Electrostatic interactions were calculated using the particle-mesh Ewald method with a cutoff of 1.2 nm. The non-bonded interaction cutoff was set to 10 Å, updated every 10 steps. The resulting trajectory was processed to remove periodic boundary effects and subsequently analyzed for metrics, including RMSD, RMSF, contact number, and hydrogen bond count.

Based on the final frame of the molecular dynamics trajectory, virtual saturation mutagenesis was performed on the top three key amino acids within the NAMPT-PB1 protein complex using FoldX 5.1 to calculate the resulting changes in inter-protein binding energy (ΔΔG). The top 20 mutations most detrimental to protein binding, along with their corresponding binding energies, were selected for presentation.

### Polymerase activity assay

To assess viral polymerase activity, HEK293T cells were co-transfected with the pHW2000 plasmids encoding PB1 wild type (WT) or PB1 (R203P), PB2, PA, and NP, along with the pPol I firefly luciferase reporter and a Renilla luciferase control plasmid. After 24 h, relative polymerase activity was measured with a Dual-Luciferase Reporter Assay System (catalog no. E1910; Promega, USA). Firefly luciferase signals were normalized to Renilla signals to control for transfection efficiency and calculate relative activity.

### Time course inhibition assay

To investigate the antiviral stage of FK866, the compound was added at three different time points: 1 h before (pre), simultaneously with (co), or 1 h after (post) H1N1 (PR8) infection. For the pre-infection treatment group, A549 cells were incubated with FK866 at 37°C for 1 h. Subsequently, the cells were washed three times with PBS and then infected with the H1N1 (PR8) virus for 1 h. Following infection, the cells were washed three times with PBS and replenished with cell culture medium containing 2% FBS. In the co-infection group, A549 cells were co-incubated simultaneously with H1N1 (PR8) and FK866 for 1 h. Subsequently, the cells were washed three times with PBS and replenished with cell culture medium containing 2% FBS. For the post-infection treatment group, A549 cells were first infected with H1N1 (PR8) for 1 h. Subsequently, the cells were washed three times with PBS, followed by the addition of cell culture medium containing 2% FBS and supplemented with FK866. For the above three groups, the IAV replication was detected using Western blotting, qPCR, and TCID_50_ assays at 24 hpi.

The antiviral period of FK866 was further investigated after IAV entry into host cells. A549 cells were first infected with H1N1 (PR8) for 1 h. Subsequently, the cells were washed three times with PBS, followed by the addition of cell culture medium containing 2% FBS and supplemented with FK866. FK866 was administered at 1, 2, 3, 4, 5, and 6 h after H1N1 (PR8) infection. The cells and culture supernatants were collected to determine viral replication using Western blotting, TCID_50_, and qPCR assays at 8 hpi.

### Animal experiments

Female 6- to 8-week-old BALB/c mice were used in the experiments from Si Pei Fu (Beijing, China). To evaluate the toxicity effects of FK866 in mice, FK866 of different concentrations (5, 10, and 20 mg/kg) was orally administered four times. Survival rates and body weight changes of mice were observed and recorded. To determine the anti-IAV effect of FK866, the drug was orally administered every other day from 7 days before to 14 days post-infection (dpi). On day 0, every mouse was intranasally infected with 4.8 × 10^4^ PFU H1N1 (PR8). The body weights and survival of the mice in each group were monitored, and lungs of the mice were collected to determine the viral load by TCID_50_ and qPCR assays.

### Histopathological analysis

For histopathology observation of lungs, the hematoxylin-eosin staining was performed according to previously described (35). Briefly, mouse tissues were fixed in 4% paraformaldehyde and embedded in paraffin. Then, 4 μm-thick sections were prepared and stained with H&E.

### Statistical analysis

Statistical analysis was performed in GraphPad Prism (version 9.5.0; San Diego, CA, USA). Statistical significance was analyzed using unpaired Student’s *t*-test and one-way ANOVA. The specific details of the statistical tests are described in the figure legends.

## RESULTS

### The NAMPT inhibitor FK866 inhibits IAV replication

**Fig 1:**
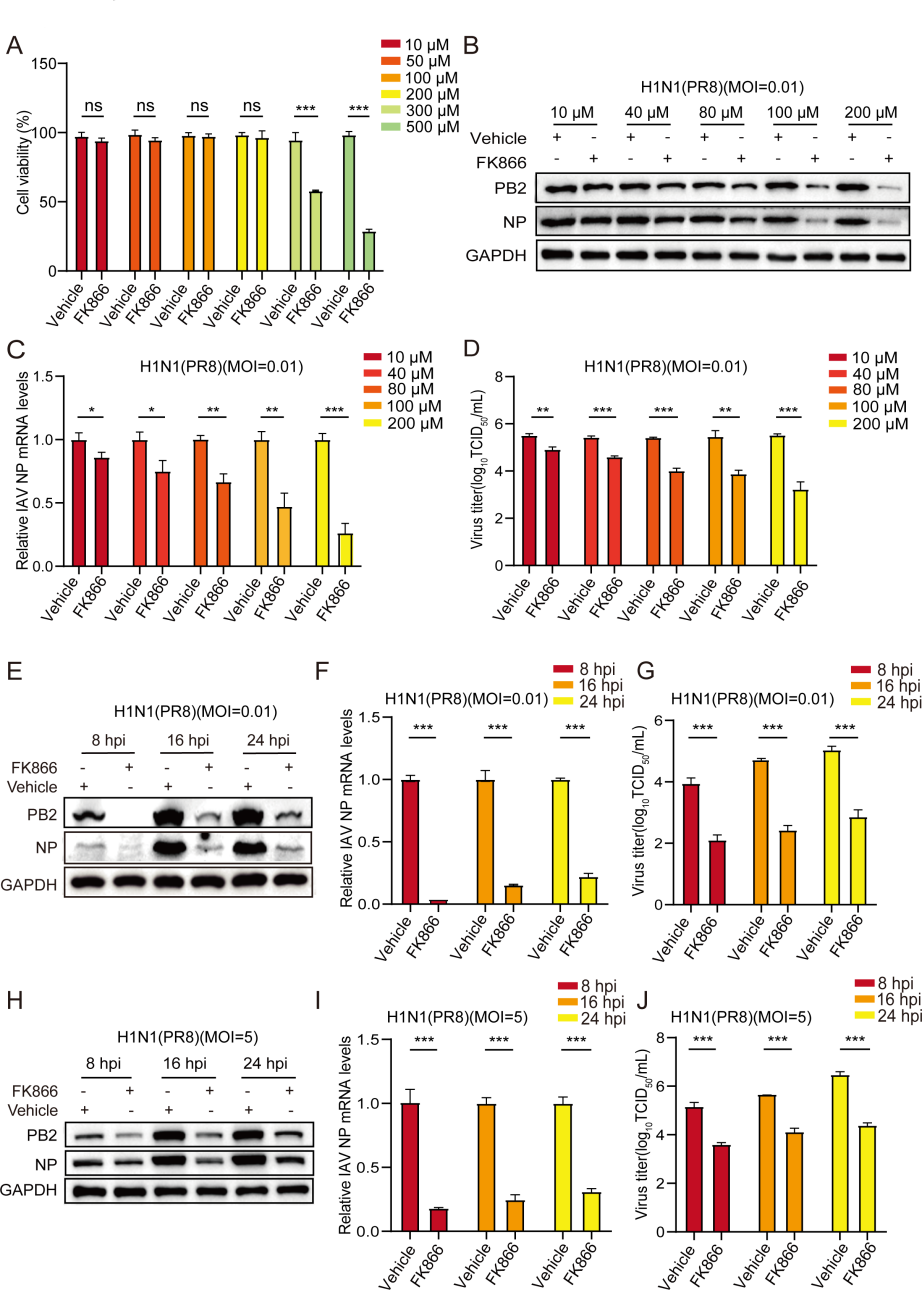
FK866 significantly inhibits IAV replication. (**A**) Cytotoxic effects of FK866 on A549 cells were assessed using CCK-8 assays after 24 h of treatment. (**B–D**) The antiviral effects of different concentrations of FK866 (10, 40, 80, 100, and 200 μM) against H1N1 (PR8) infection at a multiplicity of infection (MOI) of 0.01 in A549 cells were analyzed by Western blotting at 24 h post-infection (hpi) (**B**). NP mRNA levels and viral titer of H1N1 (PR8) were detected by qPCR (**C**) and TCID_50_ assays (**D**). (**E–G**) Western blotting was performed to detect NP and PB2 protein levels of H1N1 (PR8) at an MOI of 0.01 in FK866- and vehicle-treated cells at 8, 16, and 24 hpi (**E**). The NP mRNA levels and viral titer of H1N1 (PR8) were detected by qPCR (**F**) and TCID_50_ assays (**G**). (**H–J**) Western blotting was performed to detect NP and PB2 protein levels of H1N1 (PR8) at an MOI of 5 in FK866- and vehicle-treated cells at 8, 16, and 24 hpi (**H**). NP mRNA levels and viral titer of H1N1 (PR8) were detected by qPCR (**I**) and TCID_50_ assays (**J**). Data are presented as mean ± SD of three independent experiments. *P < 0.05, **P < 0.01, ***P < 0.001. ns, non-significant.

To assess the role of NAMPT in IAV infections, we employed a NAMPT inhibitor FK866 (Fig. S1A) (30, 36). The cytotoxicity of FK866 for A549 cells was evaluated using CCK-8 assays. At concentrations below 200 μM, FK866 exhibited no significant cytotoxicity on A549 cells (Fig. 1A). Therefore, FK866 of 10, 40, 80, 100, and 200 μM was used to examine its effect on the replication of influenza virus A/PR8/34 (H1N1, PR8). Western blotting assays showed that with the increase of FK866 concentrations, viral NP and PB2 protein levels decreased, compared to the vehicle-treated group (Fig. 1B). Consistently, as the FK866 concentration increased from 10 to 200 μM, NP mRNA levels and viral titers displayed a concentration-dependent reduction, compared to the vehicle-treated group (Fig. 1C and D).

In addition, the effect of FK866 on the IAV replication was evaluated at different time points after PR8 virus infection. Viral NP and PB2 protein levels were significantly decreased in the FK866-treated cells, compared with those in vehicle control cells at 8, 16, and 24 hpi (Fig. 1E). In line with this, NP mRNA levels and viral titers were significantly reduced in FK866-treated cells compared to the vehicle control (Fig. 1F and G). Moreover, FK866 also attenuated the replication of IAV in cells challenged with PR8 virus at a higher multiplicity of infection (5 MOI) (Fig. 1H through J). Furthermore, the combination of FK866 with oseltamivir enhanced the antiviral efficacy of oseltamivir against the IAV infection (Fig. S1B through E). These results suggest that the NAMPT inhibitor FK866 significantly suppresses the replication of IAV.

### FK866 or depletion of NAMPT suppresses the replication of various IAV subtypes

**Fig 2:**
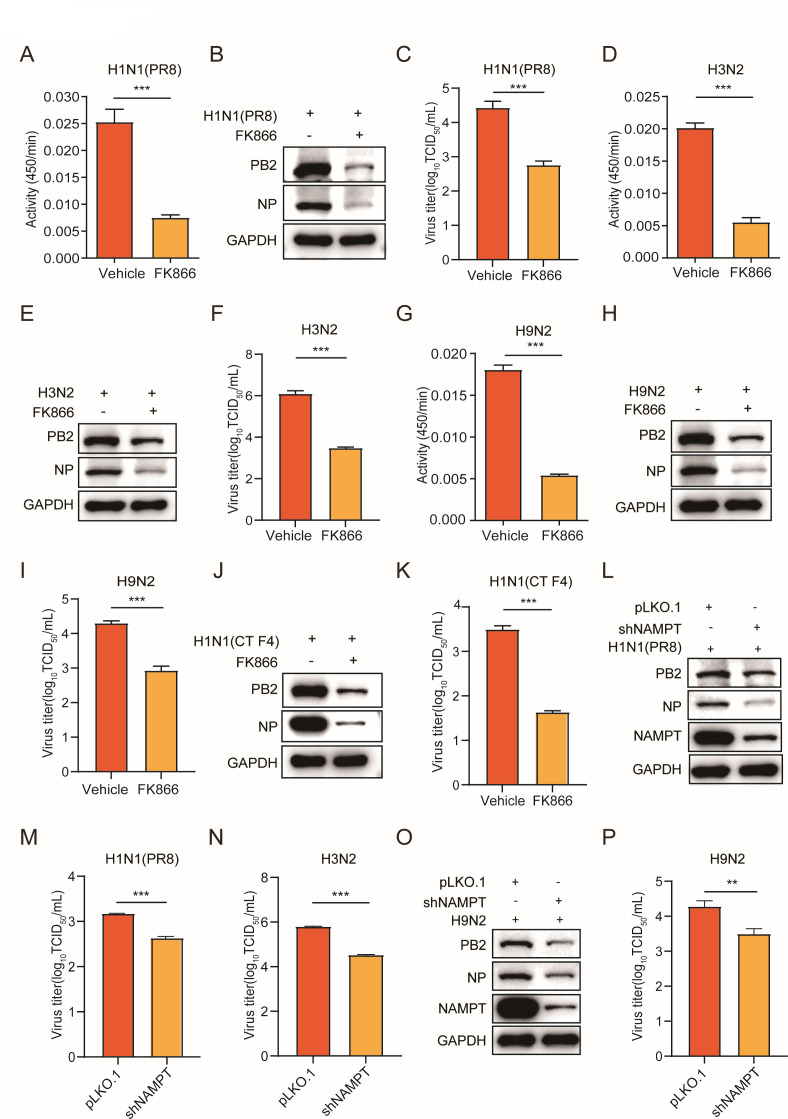
FK866 or depletion of NAMPT suppresses the replication of various IAV subtypes. (**A–C**) The NAMPT activity was measured in FK866- and vehicle-treated A549 cells infected with H1N1 (PR8) at an MOI of 0.01 for 24 h (**A**). NP and PB2 protein levels and viral titers were detected by Western blotting (**B**) and TCID_50_ assays (**C**). (**D–F**) The NAMPT activity was measured in FK866- and vehicle-treated A549 cells infected with H3N2 at an MOI of 0.01 for 24 h (**D**). NP and PB2 protein levels and viral titers were examined by Western blotting (**E**) and TCID_50_ assays (**F**). (**G–I**) The NAMPT activity was measured in FK866- and vehicle-treated A549 cells infected with H9N2 at an MOI of 0.01 for 24 h (**G**). NP and PB2 protein levels and viral titers were detected by Western blotting (**H**) and TCID_50_ assays (**I**). (**J and K**) The antiviral effect of FK866 against 0.01 MOI H1N1 (CT F4) infection in A549 cells was analyzed by Western blotting at 24 hpi (**J**). The viral titers of H1N1 (CT F4) were detected by TCID_50_ assays (**K**). (**L–P**) Control and NAMPT knockdown A549 cells were infected with H1N1 (PR8) (MOI = 0.01, 24 h) (**L and M**), H3N2 (MOI = 0.01, 24 h) (**N**), or H9N2 (MOI = 0.01, 24 h) (**O and P**). NP and PB2 protein levels, and viral titers were examined by Western blotting (**L and O**) and TCID_50_ assays (**M, N, and P**). Data are presented as mean ± SD of three independent experiments. **P < 0.01, ***P < 0.001.

Next, we evaluated the effect of FK866 on different IAV subtypes. As shown in Fig. 2A, the NAMPT activity was greatly reduced in FK866-treated cells compared with that in the vehicle-treated cells following H1N1 (PR8) infection, and accordingly, viral NP and PB2 protein levels and viral titers significantly decreased in the FK866-treated cells (Fig. 2B and C). Furthermore, we infected A549 cells with other strains of IAVs, including A/WSN/1933 (H1N1, WSN), H1N1 (CT F4), H3N2, and H9N2 subtype viruses, and similar results were observed in these experiments. NP and PB2 mRNA and protein levels and IAV titers significantly reduced in the FK866-treated cells, in which the NAMPT activity was blocked, compared to the vehicle-treated cells (Fig. 2D through K, and Fig. S2A through E). Additionally, we evaluated the effect of NAMPT deficiency on viral replication. We generated A549 cells stably expressing specific shRNA targeting NAMPT (shNAMPT) or the control (pLKO.1). Then, control and NAMPT knockdown cells were infected with IAVs (H1N1, H3N2, or H9N2), and replication of the viruses was detected. As shown in Fig. S2L through P and Fig. S2F through H, mRNA and protein levels of viral NP and PB2 and IAV titers decreased in the NAMPT knockdown cells, compared with those in control cells. In contrast, our data showed that NAMPT overexpression enhanced the IAV replication (Fig. SI through K). Moreover, we also determined the effect of FK866 on the replication of a DNA virus, PRV, and observed that FK866 hindered the replication of the virus (Fig. S2L through N). These results indicate that treatment with FK866 or loss of NAMPT represses the replication of either IAVs (H1N1, H3N2, and H9N2) or PRV.

### FK866 mitigates the IAV replication in mice

**Fig 3:**
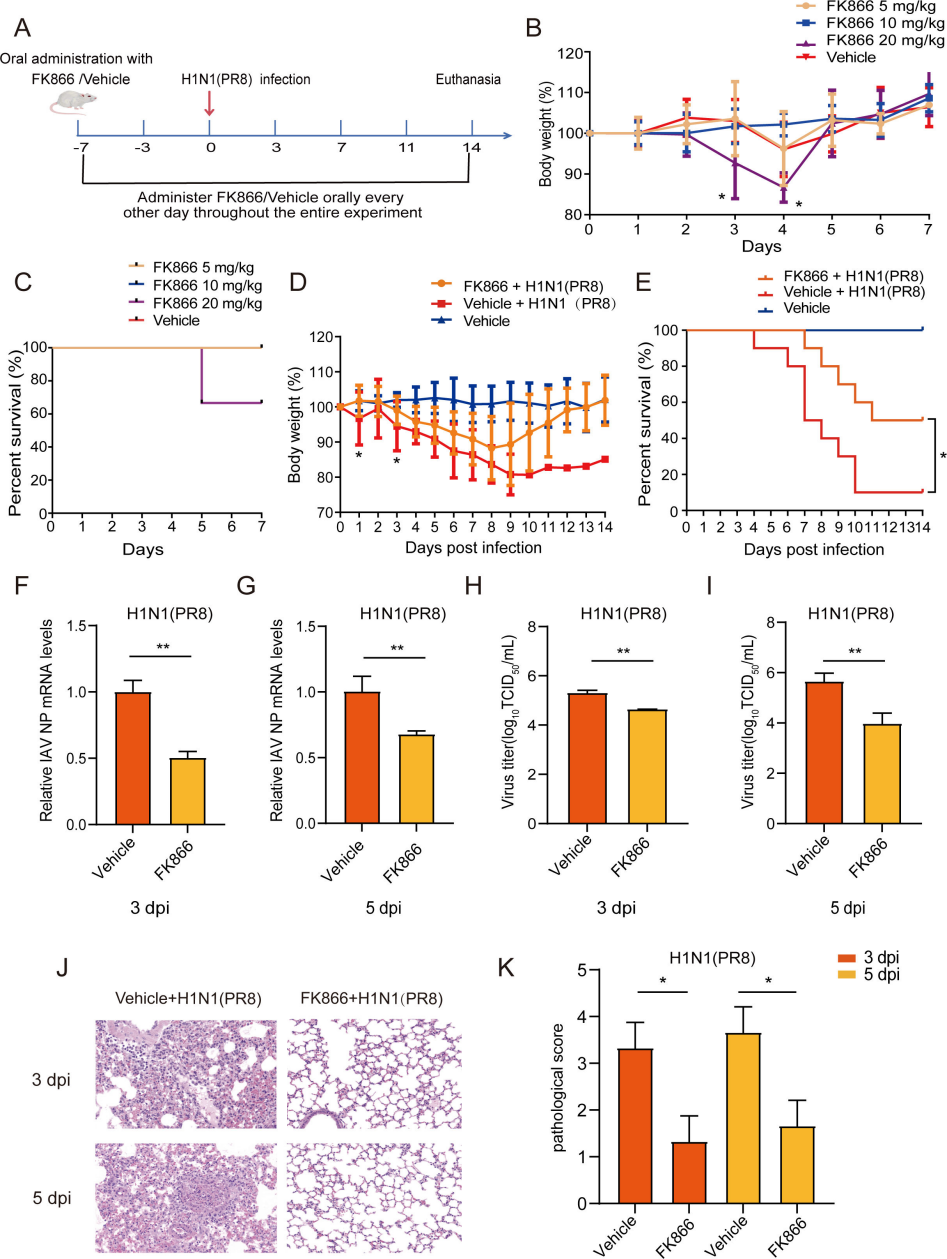
FK866 impairs the virulence of IAV in mice. (**A**) Schematic diagram of the animal experiments. FK866 was orally administered every other day for a total of 11 doses, from 7 days before infection to 14 dpi. After four doses, each mouse was intranasally administered with H1N1 (PR8) (4.8 × 10^4^ PFU). (**B and C**) The body weight loss (B) and survival rates (**C**) of mice following oral administration of FK866 (5, 10, and 20 mg/kg) or vehicle every other day for four doses over a 7-day period were monitored. (**D and E**) The body weight loss (D) and survival rates (**E**) of vehicle-treated mice (vehicle), vehicle-treated mice infected with PR8 virus (vehicle + PR8), and FK866 (10 mg/kg)-treated mice infected with PR8 virus (FK866 + PR8) were monitored. (**F–I**) Viral NP mRNA levels and virus titers in the lungs of vehicle- and FK866 (10 mg/kg)-treated mice infected with the PR8 virus were detected by qPCR (**F and G**) and TCID_50_ assays (**H and I**) at 3 and 5 dpi. (**J and K**) Histopathological changes in the lungs of vehicle- and FK866 (10 mg/kg)-treated mice infected with the PR8 virus were analyzed by hematoxylin and eosin staining at 3 and 5 dpi (**J**). Blinded analysis of lung tissue sections was performed to evaluate histopathological severity (**K**). Histological changes were scored based on pre-defined criteria as follows (0, no pathological lesions; 1, affected area ≤10%; 2, affected area >10% and ≤30%; 3, affected area >30% and ≤70%; 4, affected area >70% and ≤100%). Data are presented as mean ± SD. *P < 0.05, **P < 0.01.

An *in vivo* model of IAV infection was utilized to further probe the impact of FK866 on the viral pathogenesis (Fig. 3A), which was adapted from a previously established murine model with specific modifications (37). Mice were treated with 10 mg/kg FK866 orally every other day, initiated 1 week before infection, and maintained throughout the 14-day observation period. After taking the drug orally four times, the mice were intranasally administered the IAV PR8 virus. The toxicity effect was evaluated by oral administration of FK866 every other day from days 0 to 7 four times in mice. The data from toxicity experiments revealed that oral administration of FK866 at 5 and 10 mg/kg, rather than 20 mg/kg, did not induce significant body weight changes and reduce survival rates compared to the vehicle-treated control group (Fig. 3B and C). We further assessed the liver toxicity of FK866 in mice orally administered at 10 mg/kg. No significant differences in the expression of key liver function markers, including ALT, AST, TBIL, ALB, ALP, and γ-GT, were observed in the FK866-treated mice compared to the vehicle control group (Fig. S3A through F). These results indicate that FK866 (10 mg/kg) does not cause significant liver injury in mice. Therefore, a dose of 10 mg/kg FK866 was selected for oral administration in the subsequent antiviral analysis. We observed that FK866-treated mice displayed a slower body weight loss than vehicle-treated mice following the IAV infection (Fig. 3D). Of note, the body weights of FK866-treated mice initially decreased following PR8 virus infection but subsequently recovered with prolonged infection time and were comparable to those of vehicle-treated mice uninfected with PR8 virus on 14 dpi (Fig. 3D). Accordingly, the FK866-treated mice exhibited a 50% survival rate after PR8 virus infection, while that of mice treated with vehicle was 10% on 14 dpi (Fig. 3E). The viral replication was then examined in the vehicle- and FK866-treated mice challenged with the PR8 virus. Compared to vehicle-treated control mice, reduced IAV NP mRNA levels and viral titers were observed in the lungs of FK866-treated mice at 3 and 5 dpi (Fig. 3F through I). In line with this, the lungs of vehicle-treated mice exhibited severe inflammatory cell infiltration, significantly uneven widening of alveolar septa, and extensive tissue damage, compared with those of FK866-treated mice at 3 and 5 dpi (Fig. 3J and K), and the expression of IL-6, TNF-α, IL-1β, IFN-α, IFN-β, and IFN-γ was significantly lower in the lungs of FK866-treated mice than those in the vehicle control mice at 3 and 5 dpi (Fig. SG through J). These results suggest that FK866 treatment renders mice more resistant to the IAV infection.

### FK866 restrains IAV replication at the early stage after viral entry

**Fig 4:**
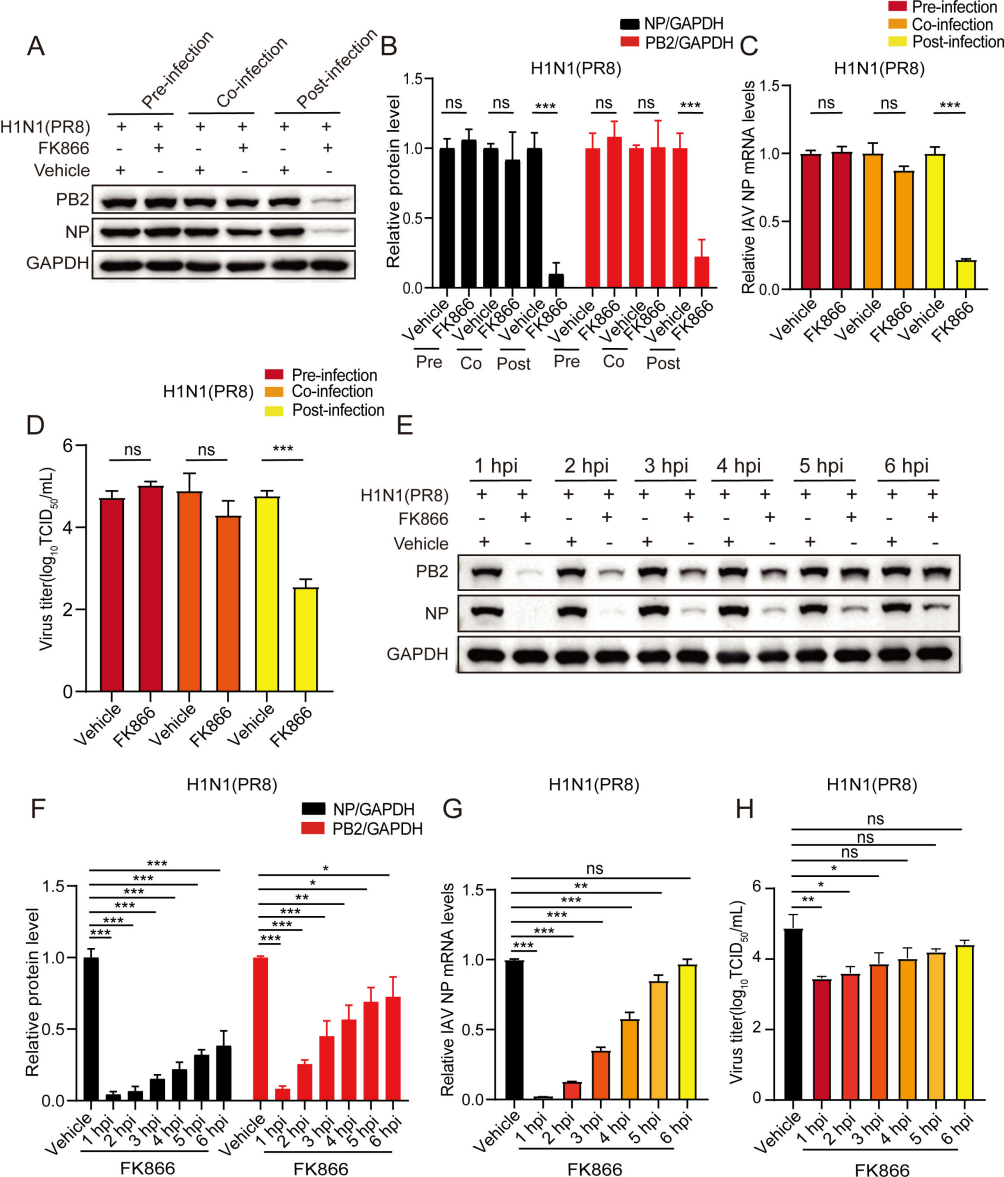
FK866 inhibits IAV replication at the early stage of viral infection. (**A–D**) Western blotting was employed to analyze the antiviral effect of FK866 in A549 cells infected with H1N1 (PR8) at an MOI of 0.01 for 24 h, when FK866 was added at the pre-infection, co-infection, and post-infection stages, respectively (**A**). The band intensities of NP and PB2 proteins were analyzed using ImageJ (B). The NP mRNA levels were detected by qPCR (**C**), and viral titers were tested by TCID_50_ assays (**D**). (**E–H**) Western blotting was performed to analyze the antiviral effect of FK866 in A549 cells infected with H1N1 (PR8) at an MOI of 0.01 for 8 h, when FK866 was added at 1, 2, 3, 4, 5, and 6 hpi, respectively (**E**). The band intensities of PB2 and NP proteins were analyzed using ImageJ (F). The NP mRNA levels were detected by qPCR (**G**). Viral titers were tested by TCID_50_ assays (**H**). Data are presented as mean ± SD of three independent experiments. *P < 0.05, **P < 0.01, ***P < 0.001. ns, non-significant.

To probe the stage at which FK866 inhibited the replication of IAV, FK866 was administered at three different time points: pre-infection, co-infection, and post-infection. As shown in Fig. 4A, when FK866 was added during post-infection, rather than pre-infection and co-infection, viral NP and PB2 protein levels were significantly decreased by 90.05% and 77.73%, respectively, in the FK866-treated cells, compared to the vehicle-treated cells (Fig. 4B). In line with this, NP mRNA levels and viral titers significantly reduced in the FK866-treated cells (Fig. 4C and D). These data imply that FK866 may play an antiviral role after the entry of IAV into host cells. Furthermore, FK866 was added at different time points after virus infection, including 1, 2, 3, 4, 5, and 6 hpi. The viral replication was then examined. We observed that FK866 exhibited potent anti-IAV activity when added at 1–3 hpi, as evidenced by the significantly reduced NP and PB2 mRNA and protein levels and decreased viral titers in the FK866-treated cells, compared with those in the vehicle-treated cells at 1 to 3 hpi (Fig. 4E through H). The data suggest that FK866 mainly functions during the early phase of IAV infection.

### FK866 or loss of NAMPT impairs IAV transcription and replication

**Fig 5:**
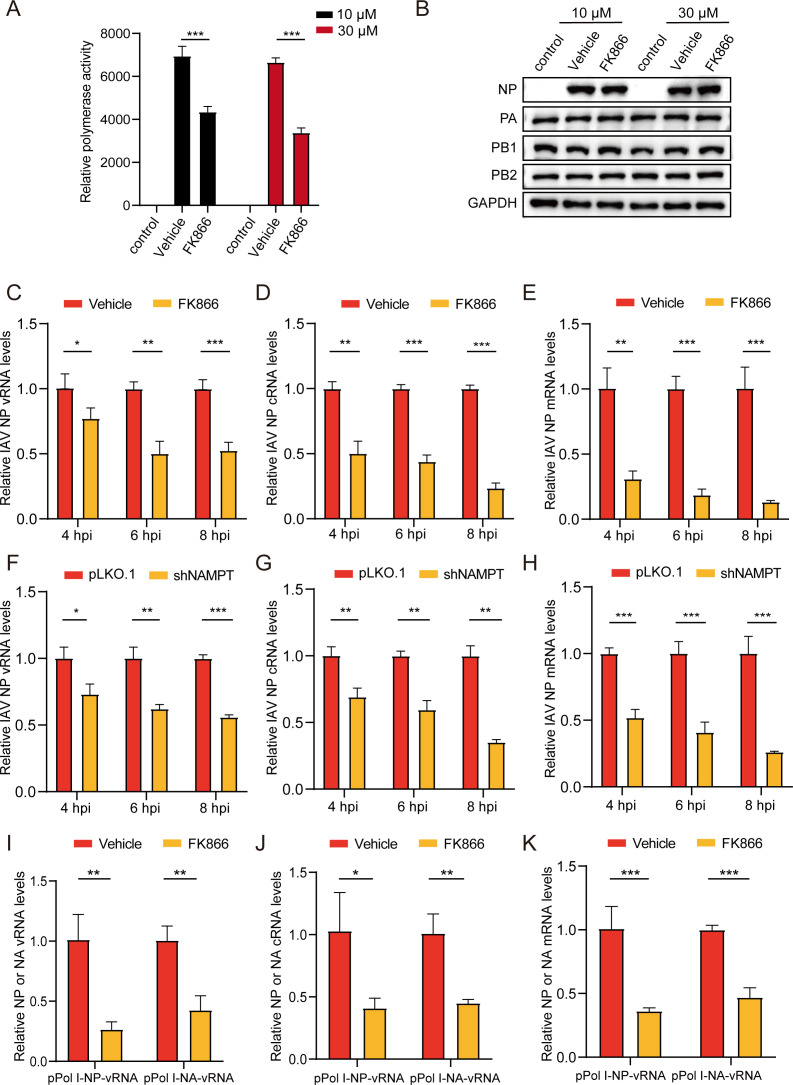
FK866 or depletion of NAMPT restricts IAV transcription and replication. (**A and B**) The viral polymerase activity in vehicle- and FK866-treated HEK293T cells transfected with PA, PB1, PB2, NP, pPol I-Luc, and Renilla luciferase expression plasmids for 24 h was examined by luciferase assays (**A**). Western blotting was performed to detect the expression of PA, PB1, PB2, and NP proteins (**B**). (**C–E**) The NP vRNA, cRNA, and mRNA levels in FK866- and vehicle-treated A549 cells infected with H1N1 (PR8) at an MOI of 5 were detected by qPCR at 4, 6, and 8 hpi. (**F–H**) NP vRNA, cRNA, and mRNA levels in control and NAMPT knockdown A549 cells infected with H1N1 (PR8) at an MOI of 5 were detected by qPCR at 4, 6, and 8 hpi. (**I–K**) HEK293T cells were transfected with either pPol I-NA-vRNA or pPol I-NP-vRNA, along with plasmids encoding PB2, PB1, PA, and NP, and then treated with FK866 or vehicle. The levels of NP or NA vRNA, cRNA, and mRNA were examined by qPCR at 24 h post-transfection. Data are presented as mean ± SD of three independent experiments. *P < 0.05, **P < 0.01, ***P < 0.001.

To determine whether FK866 exerts its antiviral effect at the early stage of IAV infection by interfering with the viral RNA synthesis, a luciferase assay to assess the vRNP activity was performed (38). HEK293T cells were transfected with the vRNP components (PB2, PB1, PA, and NP), along with a polymerase I-driven luciferase reporter flanked by NS segment termini, and exposed to FK866 or vehicle treatment. Of note, a marked reduction in the luciferase activities was detected following FK866 treatment, compared to the vehicle control (Fig. 5A and B; Fig. S4A), suggesting that FK866 compromises the vRNP activity, and may inhibit the IAV transcription and replication.

To further address the effect of FK866 on IAV transcription and replication, A549 cells were infected with the PR8 virus at an MOI of 5 and then treated with FK866 or vehicle. The NP vRNA, mRNA, and cRNA levels were measured by RT-qPCR at 4, 6, and 8 hpi. We observed that the levels of all three species of viral RNA were significantly decreased in FK866-treated cells compared with those in vehicle control cells following PR8 virus infection (Fig. 5C through E). In addition, we infected control and NAMPT knockdown A549 cells with the PR8 virus, and NP vRNA, mRNA, and cRNA levels were detected. The expression of NP mRNA, cRNA, and vRNA was markedly lower in NAMPT knockdown cells than that in the control cells post IAV infection (Fig. 5F through H), implying that NAMPT was required for efficient IAV RNA synthesis.

Next, HEK293T cells were transfected with the vRNP components (PB2, PB1, PA, and NP), along with a Pol I-driven NA or NP vRNA expression plasmid, and then incubated with FK866 or vehicle control (38, 39). The levels of NA- or NP-derived vRNA, cRNA, and mRNA examined by RT-qPCR after 24 h were significantly lower in FK866-treated cells compared with those in the vehicle control cells (Fig. 5I through K). In addition, another assay was conducted using the transcription-inactive PB2-E361A mutant (Fig. S4B and E) (38, 40). HEK293T cells were transfected with PB2-E361A, PB1, PA, and NP, along with pPol I-NA-vRNA or pPol I-NP-vRNA, and treated with FK866 or vehicle. As shown in Fig. S4C, D, F and G, FK866 significantly decreased the expression of vRNA and cRNA of NP or NA, compared to the vehicle control. Together, these results suggest that inhibition or depletion of NAMPT perturbs the vRNP activity, thereby limiting IAV transcription and replication.

### NAMPT facilitates the PB1-PA and PB1-PB2 interactions

**Fig 6:**
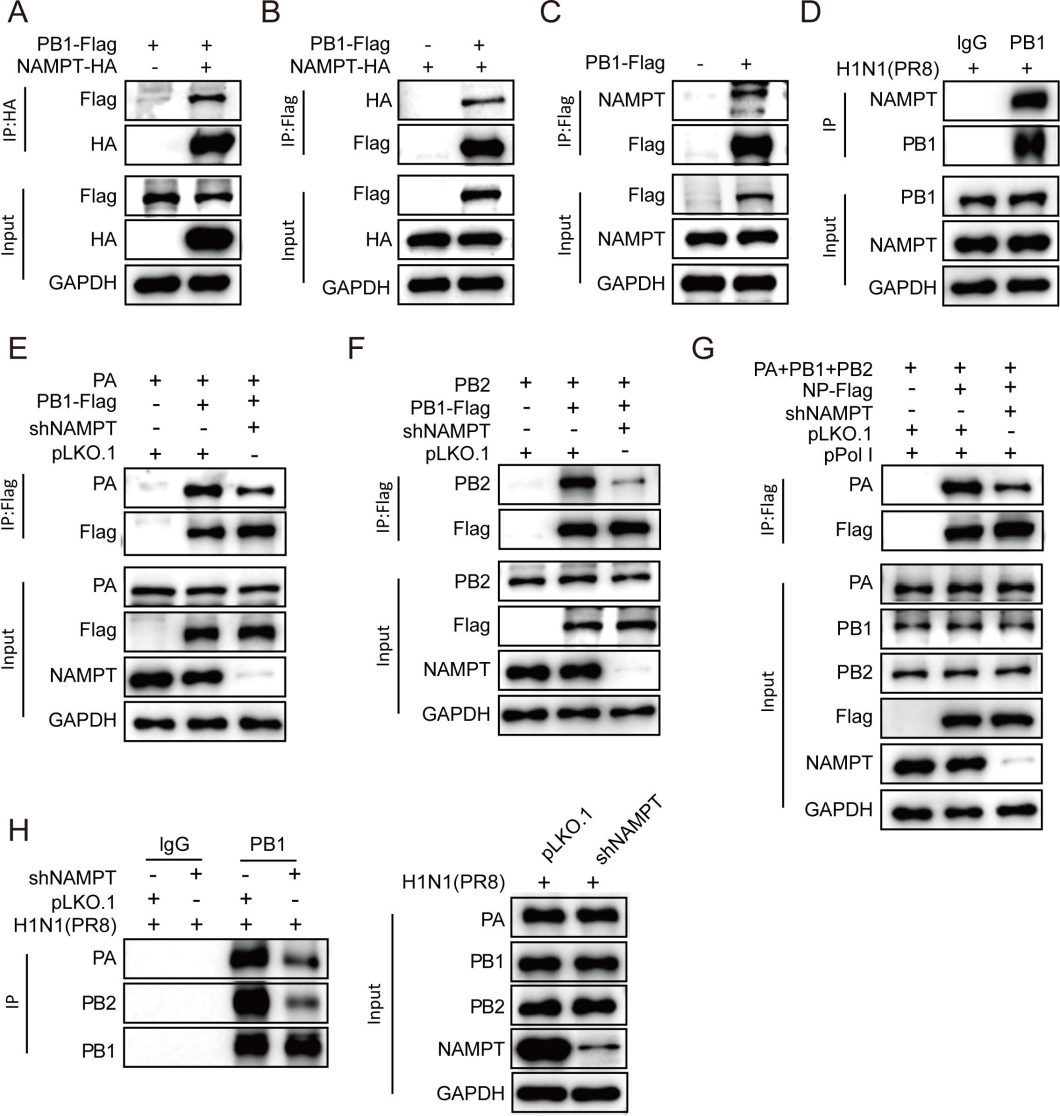
NAMPT is involved in the association of PB1 with PA and PB2. (**A**) HEK293T cells were co-transfected with PB1-Flag and NAMPT-HA plasmids, and then Co-IP was performed using NAMPT-HA as the bait protein to pull down PB1-Flag. (**B**) HEK293T cells were co-transfected with PB1-Flag and NAMPT-HA plasmids, followed by Co-IP assays using PB1-Flag as the bait protein to pull down NAMPT-HA. (**C**) HEK293T cells were transfected with PB1-Flag, and then Co-IP was performed using PB1-Flag as the bait protein to pull down endogenous NAMPT. (**D**) A549 cells were infected with H1N1 (PR8), followed by Co-IP assays using PB1 as the bait protein to pull down endogenous NAMPT. (**E**) Control and NAMPT knockdown HEK293T cells were transfected with PB1-Flag and PA plasmids. Then, PB1-Flag was immunoprecipitated as the bait protein to pull down PA. (**F**) Control and NAMPT knockdown HEK293T cells were transfected with PB2 and PB1-Flag plasmids. PB1-Flag was then immunoprecipitated as the bait protein to pull down PB2. (**G**) Control and NAMPT knockdown HEK293T cells were transfected with NP-Flag, PB1, PB2, and PA plasmids. Then, NP-Flag was immunoprecipitated as the bait protein to pull down PA. (**H**) Control and NAMPT knockdown A549 cells were infected with H1N1 (PR8). PB1 was immunoprecipitated as the bait protein to pull down PA or PB2.

Inhibition or depletion of NAMPT impairs the vRNP activity and restrains the IAV transcription and replication. This prompted us to ask whether NAMPT may regulate the vRNP activity and IAV replication via interaction with the vRNP subunits. To test this, HEK293T cells were transfected with NAMPT-HA and PB1-Flag, PB2-Flag, PA-Flag, or NP-Flag plasmids, respectively, followed by IP and Western blotting assays. We observed that IP of NAMPT-HA effectively brought down PB1-Flag or PA-Flag, but not PB2-Flag and NP-Flag, in the cell lysates (Fig. 6A; Fig. S5A through C), and consistently, reciprocal IP of Flag-PB1 or Flag-PA brought down NAMPT-HA (Fig. 6B; Fig. S5D), implying an association of NAMPT with PB1 and PA. Then, PB1-Flag was transfected into HEK293T cells, followed by IP and Western blotting assays to detect endogenous NAMPT in the cell lysates. As expected, IP of PB1-Flag clearly brought down NAMPT in the cell lysate (Fig. 6C). Furthermore, we examined the association of endogenous NAMPT with PB1 during IAV infection. A549 cells were infected with the PR8 virus and subjected to IP assays using anti-PB1 antibody with IgG as a negative control, followed by Western blotting to detect NAMPT in the cell lysates. As shown in Fig. 6D, IP of PB1 effectively pulled down endogenous NAMPT in the PR8 virus-infected cell lysates.

Next, we further addressed the effect of NAMPT on the PB1-PA and PB1-PB2 interactions. Control and NAMPT knockdown HEK293T cells were transfected with PB1-Flag and PA or PB2 plasmids respectively, followed by IP and Western blotting assays. Dramatically decreased amounts of PA or PB2 were observed in the immunoprecipitates by PB1-Flag using cell lysates derived from NAMPT knockdown cells (Fig. 6E and F), suggesting an impaired PB1-PA or PB1-PB2 interaction in the absence of NAMPT. Similarly, the PA signals retrieved by NP-Flag were significantly decreased in NAMPT knockdown cells, compared to the control cells (Fig. 6G).

Furthermore, we determined the effect of NAMPT on the association of PB1 with PA and PB2 during IAV infection. Control and NAMPT knockdown A549 cells were infected with PR8 virus and subjected to IP assays using anti-PB1 antibody with IgG as a negative control, followed by Western blotting to detect PA and PB2 in the cell lysates. As shown in Fig. 6H, the amounts of PA or PB2 IPed by PB1 were decreased in the immunoprecipitates using cell lysates derived from NAMPT knockdown cells, compared to those from control cells. Together, these results suggest that NAMPT promotes the association of PB1 with PA and PB2, thereby facilitating the assembly of the vRNP complex.

### Arginine 203 of PB1 is critical for its interaction with NAMPT

**Fig 7:**
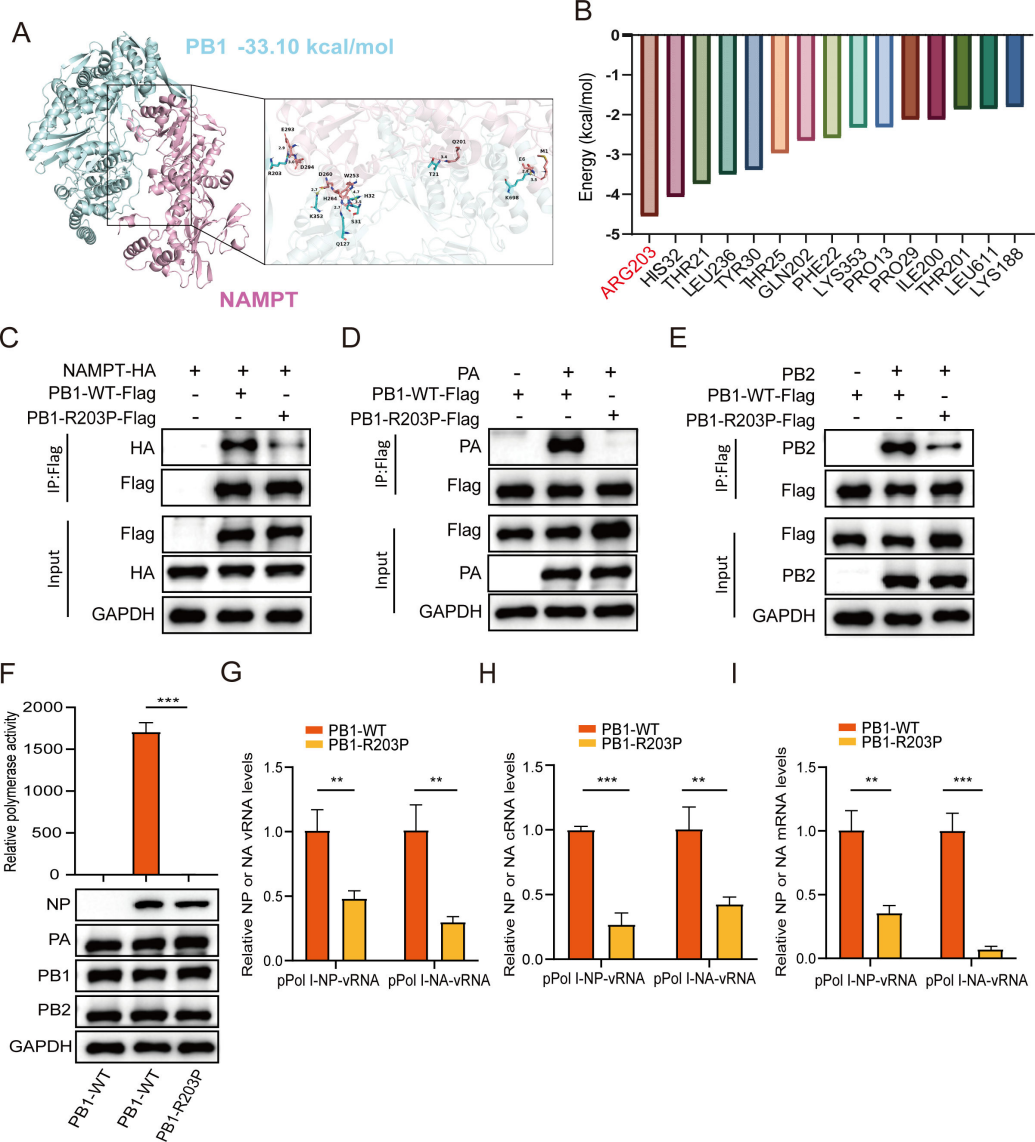
Arginine 203 of PB1 is critical for its interaction with NAMPT, PA, and PB2. (**A**) The interaction between NAMPT and PB1 was analyzed by molecular docking. (**B**) Critical amino acid residues in PB1 that mediate its interaction with NAMPT were identified through molecular dynamics analysis. (**C**) HEK293T cells were co-transfected with NAMPT-HA, along with PB1-WT-Flag or PB1-R203P-Flag, followed by Co-IP assays using the Flag-tagged protein as the bait protein to pull down NAMPT-HA. (**D**) HEK293T cells were transfected with PB1-WT-Flag or PB1-R203P-Flag, along with PA, and then Co-IP was performed using Flag-tagged protein as the bait protein to pull down PA. (**E**) HEK293T cells were transfected with PB1-WT-Flag or PB1-R203P-Flag, along with PB2. Co-IP was then performed using the Flag-tagged protein as the bait protein to pull down PB2. (**F**) HEK293T cells were transfected with PA, PB2, NP, pPol I-Luc, and Renilla luciferase expression plasmids, along with PB1-WT or PB1-R203P. The luciferase activity was measured at 24 h post-transfection. Western blotting was performed to detect the expression of PA, PB1, PB2, and NP. (**G–I**) HEK293T cells were transfected with either pPol I-NA-vRNA or pPol I-NP-vRNA, together with plasmids encoding PB2, PA, NP, and either PB1-WT or PB1-R203P. The levels of NP or NA vRNA, cRNA, and mRNA were examined by qPCR at 24 h post-transfection. Data are presented as mean ± SD of three independent experiments. **P < 0.01, ***P < 0.001.

Next, we further probed the residue of viral PB1 protein that is critical for its interaction with NAMPT. Molecular docking analysis was first performed to analyze the interaction strengths between NAMPT and viral PB1 or PA protein. As shown in Fig. 7A and Fig. S5E, the binding energy for the NAMPT-PA complex was −31.29 kcal/mol, while that for the NAMPT-PB1 complex was −33.10 kcal/mol, implying a stronger interaction between NAMPT and PB1. Thus, we focused on PB1 protein for subsequent studies. To identify the PB1 residues responsible for its interaction with NAMPT, molecular dynamics analysis was employed, which revealed that the arginine (R) 203 of PB1 exhibited the strongest binding affinity for NAMPT (Fig. 7B). Further virtual saturation mutagenesis analysis revealed that the substitution of arginine (R) 203 with proline (P) could highly disrupt the binding between PB1 and NAMPT (Table S2). Then, we transfected HEK293T cells with NAMPT-HA and PB1-WT-Flag or PB1-R203P-Flag, respectively, followed by IP assays to examine the interaction between NAMPT and PB1. Dramatically reduced amounts of NAMPT-HA were observed in the immunoprecipitates by PB1-R203P-Flag, compared with those by PB1-WT-Flag (Fig. 7C), indicating that the replacement of PB1 arginine (R) 203 with proline (P) significantly blocked the interaction between PB1 and NAMPT. In line with this, the amounts of PA and PB2 pulled down by PB1-R203P-Flag were markedly reduced compared to PB1-WT-Flag (Fig. 7D and E). These observations prompted us to further evaluate the effect of the PB1 mutation (R203P) on the vRNP activity. To test this, HEK293T cells were transfected with PA, PB1, PB2, pPol I-firefly luciferase and Renilla luciferase plasmids, along with WT or mutant PB1 (R203P), followed by luciferase assays. A significant decrease in the vRNP activity was observed in cells expressing mutant PB1 (R203P), compared to the WT PB1-expressing cells (Fig. 7F), indicating that disruption of the NAMPT-PB1 interaction impaired the IAV vRNP activity. To further delineate the effect of PB1 (R203P) on IAV transcription and replication, we transfected HEK293T cells with PB2, NP, PA, and PB1 (PB1-WT, or PB1-R203P), along with pPol I-NA-vRNA or pPol I-NP-vRNA. The expression of NA or NP vRNA, cRNA, and mRNA was detected. Significantly decreased vRNA, cRNA, and mRNA levels of NP and NA were observed in cells expressing mutant PB1 (R203P), compared with those in cells expressing WT PB1 (Fig. 7G through I). Additionally, sequence analysis showed that R203 of PB1 was conserved among different IAV subtypes (Table S3), implying a vital role of PB1 R203 in the IAV infection. Together, these results suggest that arginine 203 of PB1 is critical for its interaction with NAMPT, thereby enabling viral transcription and replication.

## DISCUSSION

IAV hijacks host cellular machinery to facilitate viral transcription and translation during the infection, with particular dependence on host factors supporting the function of the RdRp complex (41). In turn, this host-virus interaction establishes host cellular proteins as promising targets for novel antiviral drugs. Multiple host proteins have been implicated in modulating the RdRp activity, with heat shock protein 90 being the first identified participant in this process (42, 43). NAMPT, a rate-limiting enzyme of NAD^+^ synthesis, plays pleiotropic roles in cellular physiology (23). FK866, a specific NAMPT inhibitor, is known to induce tumor cell apoptosis (44). In this study, we found that FK866 effectively inhibited the IAV replication *in vitro* and *in vivo*. We selected A549 cells as the host cell model and found that FK866 exhibited no significant cytotoxicity at concentrations up to 200 μM. IAVs have evolved numerous drug-resistant variants due to their highly mutable genomes, leading to the current limitation that most antiviral drugs are effective against only specific strains but lack broad-spectrum activity against different subtypes of IAVs (45, 46). Our results show that FK866 significantly inhibits the replication of multiple subtypes of IAVs, including H1N1, H3N2, and H9N2, suggesting its potential as a broad-spectrum antiviral agent. Additionally, we observed that FK866-treated mice exhibited significant resistance to IAV infection, as evidenced by a lower degree of tissue injury, slower body weight loss, and better survival, than untreated animals challenged with IAV. Of note, the mice received oral administration of FK866 rather than injection, facilitating conveniently future clinical applications. *In vivo* experiments revealed that FK866 inhibited IAV replication but provided only 50% protection, which may be attributed to the administration route of FK866. The inhibitor is absorbed and passes systemic circulation to reach lungs, thereby exerting antiviral effects, during which partial degradation or clearance may occur. Nanoparticles, as effective delivery carriers, can improve drug solubility, enhance bioavailability, prolong drug release duration, and offer advantages such as targeted delivery and reduced side effects (47, 48). Future studies are deserved to explore the potential of coupling FK866 with nanoparticles to improve its antiviral efficacy.

The replication of IAV involves five critical stages: receptor-mediated attachment, invasion, uncoating, viral protein synthesis, and virion assembly and release, with each stage during the viral life cycle representing a potential target for antiviral intervention (49, 50). Our data indicate that FK866 exhibits no antiviral activity when administered prior to IAV infection or viral entry. In contrast, the addition of FK866 post-virus infection showed significant antiviral effects, suggesting that FK866 does not interfere with the viral attachment and entry but instead inhibits subsequent stages of the viral replication, particularly viral protein synthesis. The IAV life cycle is completed within 8 h (49, 51). Our results showed that FK866 significantly inhibited the viral replication when administered at 1–3 hpi but exhibited minimal inhibitory effects when added at 4–6 hpi. These observations indicate that FK866 exhibits markedly antiviral activity at the early stages of IAV infection, during which the viral replication highly depends on the early viral proteins, particularly the vRNP complex (PA, PB1, PB2, and NP) and non-structural proteins, whose functional impairment could effectively suppress the replication of IAV (20, 52). Indeed, our data showed that FK866 restrained the activity of the vRNP complex, thereby suppressing the synthesis of IAV vRNA, cRNA, and mRNA. To gain insight into the mechanism underlying inhibition of the vRNP activity by FK866, we examined the interaction between NAMPT and the vRNP subunits and found that NAMPT interacted with the viral PB1 protein. Furthermore, we identified that arginine 203 of PB1 was critical for its interaction with NAMPT. The mutation (R203P) of PB1 impeded its association with NAMPT, PA, and PB2, thereby restraining the vRNP activity and repressing IAV transcription and replication. This is consistent with a previous study showing that mutation of PB1 arginine 203 to alanine disturbed the viral polymerase activity (53). Of note, our data indicated that NAMPT also interacted with the viral PA protein. Further studies are needed to map specific domains and residues responsible for the interaction of NAMPT, PB1, and PA. The precise mechanisms by which NAMPT regulates the vRNP activity during IAV infection remain to be further determined.

Given the crucial roles of NAMPT in cellular pathophysiological processes, it represents a promising target for developing novel antitumor agents (44). Previous studies have revealed that FK866 binds to NAMPT through hydrogen bonds with Asp219, Ser241, Val242, and Ser275 and hydrophobic interactions with Arg349. These molecular interactions impair NAMPT enzymatic activity (54). Viral infections can reprogram cellular metabolic pathways to manipulate essential nutrients and energy for their own replication (55). For instance, SARS-CoV-2 reprograms glucose metabolism to facilitate its infection (47). Human immunodeficiency virus and porcine reproductive and respiratory syndrome virus can modulate lipid metabolism to antagonize host NF-κB or IFN-β signaling pathways (48, 56). Additionally, amino acid metabolism also has been reported to contribute to viral immune evasion. Newcastle disease virus regulates glutamine and the transporter SLC1A3 to benefit its infection (57). Consequently, targeting these metabolism-related molecules or pathways represents a promising strategy for developing antiviral drugs. However, such metabolic interventions may disrupt normal cellular physiology and alter cell states (58). Further in-depth studies are warranted to evaluate the efficacy and safety of targeting key metabolic molecules or pathways for antiviral research. Moreover, FK866 can induce tumor cell apoptosis, thereby exerting antitumor effects. During viral infection, the host activates apoptosis-related signaling pathways (59), and apoptosis plays a crucial role in the viral replication (60, 61). Whether FK866 may inhibit the IAV replication by modulating apoptosis signaling pathways and the underlying mechanisms warrants further investigation.

Collectively, our studies demonstrate that the NAMPT inhibitor FK866 inhibits the replication of various IAV subtypes (H1N1, H3N2, and H9N2). Moreover, FK866 interferes with the viral polymerase activity, thereby restricting IAV transcription and replication. These findings reveal a critical role of NAMPT in regulating IAV replication and suggest that targeting NAMPT may be a potential antiviral strategy against IAV infection.

## Data Availability

All data supporting the findings of this study are available within the article and its supplementary materials. The protein sequences used for homology modeling were retrieved from the UniProt Knowledgebase under the following accession numbers: P03433, influenza A virus (strain A/Puerto Rico/8/1934 H1N1) PA (https://www.uniprot.org/uniprotkb/P03433/entry); P03431, influenza A virus (strain A/Puerto Rico/8/1934 H1N1) PB1 (https://www.uniprot.org/uniprotkb/P03431/entry); and P43490, human NAMPT (https://www.uniprot.org/uniprotkb/P43490/entry). The database reference and the specific entry citation are included in the reference list. No new data sets or database accessions were generated during the modeling process. The software and parameters used for structural modeling are described in detail within the Materials and Methods section. Any additional information related to this study is available from the corresponding author upon reasonable request.

## SUPPLEMENTAL MATERIAL

Supplemental figures jvi.00591-26-s0001.pdf Fig. S1 to S5.

Table S1 jvi.00591-26-s0002.xlsx qPCR and PCR primer sequences.

Table S2 jvi.00591-26-s0003.xlsx Virtual saturation mutation at PB1 R203 site.

Table S3 jvi.00591-26-s0004.xlsx Conservation of PB1 R203 among different IAV subtypes.

ASM does not own the copyrights to Supplemental Material that may be linked to, or accessed through, an article. The authors have granted ASM a non-exclusive, world-wide license to publish the Supplemental Material files. Please contact the corresponding author directly for reuse.

## References

[1] Zou J, Jiang M, Xiao R, Sun H, Liu H, Peacock T, Tu S, Chen T, Guo J, Zhao Y, Barclay W, Xie S, Zhou H. 2025. GGCX promotes Eurasian avian-like H1N1 swine influenza virus adaption to interspecies receptor binding. Nat Commun 16. doi:10.1038/s41467-025-55903-0 PMC11733290 39809757

[2] Spangler A, Shimberg GD, Mantus GE, Malek R, Cominsky LY, Tsybovsky Y, Li N, Gillespie RA, Ravichandran M, Creanga A, Raab JE, Gajjala SR, Mendoza F, Houser KV, Dropulic L, McDermott AB, Kanekiyo M, Andrews SF. 2025. Early influenza virus exposure shapes the B cell response to influenza vaccination in individuals 50 years later. Immunity 58:728–744. doi:10.1016/j.immuni.2025.02.004 40023164 PMC11979964

[3] Yasuhara A, Yamayoshi S, Kiso M, Sakai-Tagawa Y, Okuda M, Kawaoka Y. 2022 A broadly protective human monoclonal antibody targeting the sialidase activity of influenza A and B virus neuraminidases. Nat Commun 13. doi:10.1038/s41467-022-34521-0 PMC9632566 36329075

[4] Kumari R, Sharma SD, Kumar A, Ende Z, Mishina M, Wang Y, Falls Z, Samudrala R, Pohl J, Knight PR, Sambhara S. 2023. Antiviral approaches against influenza virus. Clin Microbiol Rev 36:e0004022. doi:10.1128/cmr.00040-22 36645300 PMC10035319

[5] Chen B, Guo G, Wang G, Zhu Q, Wang L, Shi W, Wang S, Chen Y, Chi X, Wen F, Maarouf M, Huang S, Yang Z, Chen J-L. 2024. ATG7/GAPLINC/IRF3 axis plays a critical role in regulating pathogenesis of influenza A virus. PLoS Pathog 20:e1011958. doi:10.1371/journal.ppat.1011958 38227600 PMC10817227

[6] Li Y, Liu S, Chen Y, Chen B, Xiao M, Yang B, Rai KR, Maarouf M, Guo G, Chen J-L. 2022. Syk facilitates influenza A virus replication by restraining innate immunity at the late stage of viral infection. J Virol 96:e0020022. doi:10.1128/jvi.00200-22 35293768 PMC9006912

[7] Han AX, de Jong SPJ, Russell CA. 2023. Co-evolution of immunity and seasonal influenza viruses. Nat Rev Microbiol 21:805–817. doi:10.1038/s41579-023-00945-8 37532870

[8] Chaisri U, Chaicumpa W. 2018. Evolution of therapeutic antibodies, influenza virus biology, influenza, and influenza immunotherapy. Biomed Res Int 2018:1–23. doi:10.1155/2018/9747549 PMC5994580 29998138

[9] Ni Z, Wang J, Yu X, Wang Y, Wang J, He X, Li C, Deng G, Shi J, Kong H, Jiang Y, Chen P, Zeng X, Tian G, Chen H, Bu Z. 2024. Influenza virus uses mGluR2 as an endocytic receptor to enter cells. Nat Microbiol 9:1764–1777. doi:10.1038/s41564-024-01713-x 38849624 PMC11222159

[10] Chen Z, Cai M, Chai L, Li X, Wen R, Jia J, Li H, Yu F. 2025. Anti-influenza activity of Blumea Balsamifera (L.) DC. Extract: In vitro and in vivo evaluation against multiple influenza virus strains. Virus Res 359:199606. doi:10.1016/j.virusres.2025.199606 40695408 PMC12320165

[11] Neumann G, Chen H, Gao GF, Shu Y, Kawaoka Y. 2010. H5N1 influenza viruses: outbreaks and biological properties. Cell Res 20:51–61. doi:10.1038/cr.2009.124 19884910 PMC2981148

[12] Liu Y, Deng S, Ren S, Tam RC-Y, Liu S, Zhang AJ, To KK-W, Yuen K-Y, Chen H, Wang P. 2025. Intranasal influenza virus-vectored vaccine offers protection against clade 2.3.4.4b H5N1 infection in small animal models. Nat Commun 16:3133. doi:10.1038/s41467-025-58504-z 40169649 PMC11962148

[13] Guthmiller JJ, Han J, Li L, Freyn AW, Liu STH, Stovicek O, Stamper CT, Dugan HL, Tepora ME, Utset HA, Bitar DJ, Hamel NJ, Changrob S, Zheng N-Y, Huang M, Krammer F, Nachbagauer R, Palese P, Ward AB, Wilson PC. 2021. First exposure to the pandemic H1N1 virus induced broadly neutralizing antibodies targeting hemagglutinin head epitopes. Sci Transl Med 13:eabg4535. doi:10.1126/scitranslmed.abg4535 34078743 PMC10173203

[14] Restori KH, Weaver V, Patel DR, Merrbach GA, Septer KM, Field CJ, Bernabe MJ, Kronthal EM, Minns A, Lindner SE, Lakdawala SS, Le Sage V, Sutton TC. 2025. Preexisting immunity to the 2009 pandemic H1N1 virus reduces susceptibility to H5N1 infection and disease in ferrets. Sci Transl Med 17:eadw4856. doi:10.1126/scitranslmed.adw4856 40700518 PMC12896132

[15] Wandzik JM, Kouba T, Karuppasamy M, Pflug A, Drncova P, Provaznik J, Azevedo N, Cusack S. 2020. A structure-based model for the complete transcription cycle of influenza polymerase. Cell 181:877–893. doi:10.1016/j.cell.2020.03.061 32304664

[16] Peng Q, Liu Y, Peng R, Wang M, Yang W, Song H, Chen Y, Liu S, Han M, Zhang X, Wang P, Yan J, Zhang B, Qi J, Deng T, Gao GF, Shi Y. 2019. Structural insight into RNA synthesis by influenza D polymerase. Nat Microbiol 4:1750–1759. doi:10.1038/s41564-019-0487-5 31209309

[17] te Velthuis AJW, Oymans J. 2018. Initiation, elongation, and realignment during influenza virus mRNA synthesis. J Virol 92. doi:10.1128/JVI.01775-17 PMC5774887 29142123

[18] Zhang L, Yang Q, Shao Y, Ding S, Guo J, Gao GF, Deng T. 2025. Influenza A virus NS2 protein acts on vRNA-resident polymerase to drive the transcription to replication switch. Nucleic Acids Res 53:gkaf027. doi:10.1093/nar/gkaf027 39878213 PMC11775585

[19] Bonazza S, Turkington HL, Sukumar S, Peate E, Coutts HL, Montgomery JJ, Hawthorn C, Getty EMPE, Touzelet O, Barabas J, Power UF, Courtney DG. 2026. Myoferlin is a component of late-stage vRNP trafficking vesicles for enveloped RNA viruses. Nat Commun 17:2507. doi:10.1038/s41467-026-69386-0 41654495 PMC12996415

[20] Alemany C, Da Graça J, Giai Gianetto Q, Dupont M, Paisant S, Douché T, Isel C, Delevoye C, Danglot L, Matondo M, Morel E, Brault J-B, Naffakh N. 2025. Influenza A virus induces PI4P production at the endoplasmic reticulum in an ATG16L1-dependent manner to promote the egress of viral ribonucleoproteins. PLoS Biol 23:e3002958. doi:10.1371/journal.pbio.3002958 40668844 PMC12286409

[21] Sun L, Guo X, Yu M, Wang X-F, Ren H, Wang X. 2024. Human ANP32A/B are SUMOylated and utilized by avian influenza virus NS2 protein to overcome species-specific restriction. Nat Commun 15:10805. doi:10.1038/s41467-024-55034-y 39737943 PMC11686252

[22] Liu S, Mok BW-Y, Deng S, Liu H, Wang P, Song W, Chen P, Huang X, Zheng M, Lau S-Y, Cremin CJ, Tam C-Y, Li B, Jiang L, Chen Y, Yuen K-Y, Chen H. 2021. Mammalian cells use the autophagy process to restrict avian influenza virus replication. Cell Rep 35:109213. doi:10.1016/j.celrep.2021.109213 34107256

[23] Feng S, Xie N, Liu Y, Qin C, Savas AC, Wang T-Y, Li S, Rao Y, Shambayate A, Chou T-F, Brenner C, Huang C, Feng P. 2024. Cryptic phosphoribosylase activity of NAMPT restricts the virion incorporation of viral proteins. Nat Metab 6:2300–2318. doi:10.1038/s42255-024-01162-0 39572750 PMC12085970

[24] Wang Y, Wang F, Wang L, Qiu S, Yao Y, Yan C, Xiong X, Chen X, Ji Q, Cao J, Gao G, Li D, Zhang L, Guo Z, Wang R, Wang H, Fan G. 2021. NAD+ supplement potentiates tumor killing function by rescuing defective tubby-mediated NAMPT transcription in tumor infiltrated T Cells. Cell Rep 36:109516. doi:10.1016/j.celrep.2021.109516 34380043

[25] Navas LE, Carnero A. 2021. NAD+ metabolism, stemness, the immune response, and cancer. Sig Transduct Target Ther 6:2. doi:10.1038/s41392-020-00354-w PMC7775471 33384409

[26] Izadpanah A, Mudd JC, Garcia JGN, Srivastav S, Abdel-Mohsen M, Palmer C, Goldman AR, Kolls JK, Qin X, Rappaport J. 2023. SARS-CoV-2 infection dysregulates NAD metabolism. Front Immunol 14. doi:10.3389/fimmu.2023.1158455 PMC10344451 37457744

[27] Kassegn TA, Tian Z, Du J, Niu Q, Yang J, Hongge Z, Song Y, Chai F, Zhang Z, Guan G, Yin H. 2026. NAD^+^ homeostasis attenuates Japanese encephalitis virus infection progression. FASEB J 40:e71526. doi:10.1096/fj.202503827R 41693302 PMC12907744

[28] Chen X-Y, Zhang H-S, Wu T-C, Sang W-W, Ruan Z. 2013. Down-regulation of NAMPT expression by miR-182 is involved in Tat-induced HIV-1 long terminal repeat (LTR) transactivation. Int J Biochem Cell Biol 45:292–298. doi:10.1016/j.biocel.2012.11.002 23153509

[29] Guo H-J, Li H-Y, Chen Z-H, Zhou W-J, Li J-J, Zhang J-Y, Wang J, Luo X-Y, Zeng T, Shi Z, Mo C-F. 2021. NAMPT promotes hepatitis B virus replication and liver cancer cell proliferation through the regulation of aerobic glycolysis. Oncol Lett 21:390. doi:10.3892/ol.2021.12651 33777213 PMC7988713

[30] Ye C, Mi M, Shi S, Qi L, Weng S, Wang L, Lu Y, Chen C, Tan Y, Yang M, Guo C, Bai R, Fang X, Wang J, Yuan Y. 2025. ROS‐responsive hydrogel for localized delivery of nampt and stat3 inhibitors exhibits synergistic antitumor effects in colorectal cancer through ferroptosis induction and immune microenvironment remodeling. Adv Sci (Weinh) 12. doi:10.1002/advs.202506599 PMC12412517 40492508

[31] Ogino Y, Sato A, Uchiumi F, Tanuma S-I. 2019. Genomic and tumor biological aspects of the anticancer nicotinamide phosphoribosyltransferase inhibitor FK866 in resistant human colorectal cancer cells. Genomics 111:1889–1895. doi:10.1016/j.ygeno.2018.12.012 30582964

[32] Zhou J, Li S, Hu Z, Huang S, Yang Y, Zhou C, Zhang S, Luo K, Wang C, Qing C, Zeng Z. 2025. Dendrobine attenuates lipopolysaccharide-induced acute lung injury by modulating FAM134B-mediated endoplasmic reticulum autophagy and mitochondrial function. Phytomedicine 144:156952. doi:10.1016/j.phymed.2025.156952 40532488

[33] Zheng Q, Wang Y, Liu Q, Dong X, Xie Z, Liu X, Gao W, Bai X, Li Z. 2020. FK866 attenuates sepsis-induced acute lung injury through c-jun-N-terminal kinase (JNK)-dependent autophagy. Life Sci (1962) 250:117551. doi:10.1016/j.lfs.2020.117551 32179075

[34] Bateman A, Martin M-J, Orchard S, Magrane M, Ahmad S, Alpi E, Bowler-Barnett EH, Britto R, Bye-A-Jee H, Cukura A, et al.. 2023. UniProt: the universal protein knowledgebase in 2023. Nucleic Acids Res 51:D523–D531. doi:10.1093/nar/gkac1052 36408920 PMC9825514

[35] Lv C, Yang J, Zhao L, Zou Z, Kang C, Zhang Q, Wu C, Yang L, Cheng C, Zhao Y, Liao Q, Hu X, Li C, Sun X, Jin M. 2023. Bacillus subtilis partially inhibits African swine fever virus infection in vivo and in vitro based on its metabolites arctiin and genistein interfering with the function of viral topoisomerase II. J Virol 97:e0071923. doi:10.1128/jvi.00719-23 37929962 PMC10688316

[36] Nakahata Y, Sahar S, Astarita G, Kaluzova M, Sassone-Corsi P. 2009. Circadian control of the NAD+ salvage pathway by CLOCK-SIRT1. Science 324:654–657. doi:10.1126/science.1170803 19286518 PMC6501775

[37] Lv C, Wang S, Jin Z, Zhang J, Peng Y, Chen J. 2026. Garcinone C inhibits pseudorabies virus replication through EGF/PI3K/Akt axis. Front Cell Infect Microbiol 15:1722752. doi:10.3389/fcimb.2025.1722752 41704642 PMC12907380

[38] Chen D, Zhao G, Zhou J, Sun P, He S, Lv C, Chen Y, Zhu S, Gao M, Guo G. 2026. Delactylation of viral proteins by SIRT1 suppresses influenza A virus replication. mBio 17:e0248925. doi:10.1128/mbio.02489-25 41848315 PMC13059713

[39] Wang Q, Xu S, Shen W, Wei Y, Han L, Wang Z, Yu Y, Liu M, Liu J, Deng G, Chen H, Zhu Q. 2025. N 6-methyladnosine of vRNA facilitates influenza A virus replication by promoting the interaction of vRNA with polymerase proteins. Proc Natl Acad Sci USA 122:e2411554122. doi:10.1073/pnas.2411554122 40073063 PMC11929389

[40] Kleinehr J, Schöfbänker M, Daniel K, Günl F, Mohamed FF, Janowski J, Brunotte L, Boergeling Y, Liebmann M, Behrens M, Gerdemann A, Klotz L, Esselen M, Humpf H-U, Ludwig S, Hrincius ER. 2023. Glycolytic interference blocks influenza A virus propagation by impairing viral polymerase-driven synthesis of genomic vRNA. PLoS Pathog 19:e1010986. doi:10.1371/journal.ppat.1010986 37440521 PMC10343032

[41] Bonazza S, Courtney DG. 2025. Influenza A virus RNA localisation and the interceding trafficking pathways of the host cell. PLoS Pathog 21:e1013090. doi:10.1371/journal.ppat.1013090 40267083 PMC12017568

[42] Cao M, Wei C, Zhao L, Wang J, Jia Q, Wang X, Jin Q, Deng T. 2014. DnaJA1/Hsp40 is co-opted by influenza A virus to enhance its viral RNA polymerase activity. J Virol 88:14078–14089. doi:10.1128/JVI.02475-14 25253355 PMC4249161

[43] Momose F, Naito T, Yano K, Sugimoto S, Morikawa Y, Nagata K. 2002. Identification of Hsp90 as a stimulatory host factor involved in influenza virus RNA synthesis. J Biol Chem 277:45306–45314. doi:10.1074/jbc.M206822200 12226087

[44] Gerner RR, Klepsch V, Macheiner S, Arnhard K, Adolph TE, Grander C, Wieser V, Pfister A, Moser P, Hermann-Kleiter N, Baier G, Oberacher H, Tilg H, Moschen AR. 2018. NAD metabolism fuels human and mouse intestinal inflammation. Gut 67:1813–1823. doi:10.1136/gutjnl-2017-314241 28877980 PMC6145287

[45] Lampejo T. 2020. Influenza and antiviral resistance: an overview. Eur J Clin Microbiol Infect Dis 39:1201–1208. doi:10.1007/s10096-020-03840-9 32056049 PMC7223162

[46] Pascua PNQ, Chesnokov A, Nguyen HT, Di H, La Cruz JD, Jang Y, Ivashchenko AA, Ivachtchenko AV, Karlsson EA, Sar B, Savuth C, Uyeki TM, Davis CT, Gubareva LV. 2025. Antiviral susceptibility of influenza A(H5N1) clade 2.3.2.1c and 2.3.4.4b viruses from humans, 2023–2024. Emerg Infect Dis 31:751–760. doi:10.3201/eid3104.241820 40064473 PMC11950254

[47] Nobs SP, Kolodziejczyk AA, Adler L, Horesh N, Botscharnikow C, Herzog E, Mohapatra G, Hejndorf S, Hodgetts R-J, Spivak I, et al.. 2023. Lung dendritic-cell metabolism underlies susceptibility to viral infection in diabetes. Nature 624:645–652. doi:10.1038/s41586-023-06803-0 38093014 PMC10733144

[48] Potula R, Ramirez SH, Knipe B, Leibhart J, Schall K, Heilman D, Morsey B, Mercer A, Papugani A, Dou H, Persidsky Y. 2008. Peroxisome proliferator-activated receptor-γ activation suppresses HIV-1 replication in an animal model of encephalitis. AIDS 22:1539–1549. doi:10.1097/QAD.0b013e3283081e08 18670212 PMC2688810

[49] Hutchinson EC. 2018. Influenza Virus. Trends Microbiol 26:809–810. doi:10.1016/j.tim.2018.05.013 29909041

[50] Müller M, Lauster D, Wildenauer HHK, Herrmann A, Block S. 2019. Mobility-based quantification of multivalent virus-receptor interactions: new insights into influenza A virus binding mode. Nano Lett 19:1875–1882. doi:10.1021/acs.nanolett.8b04969 30719917

[51] Chauhan RP, Gordon ML. 2022. An overview of influenza A virus genes, protein functions, and replication cycle highlighting important updates. Virus Genes 58:255–269. doi:10.1007/s11262-022-01904-w 35471490

[52] Zhang B, Xu S, Liu M, Wei Y, Wang Q, Shen W, Lei C-Q, Zhu Q. 2023. The nucleoprotein of influenza A virus inhibits the innate immune response by inducing mitophagy. Autophagy 19:1916–1933. doi:10.1080/15548627.2022.2162798 36588386 PMC10283423

[53] Uchikubo-Kamo T, Ishimoto N, Umezawa H, Hirohama M, Ono M, Kawabata H, Kamata K, Ohki M, Yoshida H, Park J-H, Tame JRH, Kawaguchi A, Park S-Y. 2024. Structural insights into Influenza A virus RNA polymerase PB1 binding to nuclear import host factor RanBP5. Biochem Biophys Res Commun 739:150952. doi:10.1016/j.bbrc.2024.150952 39536408

[54] Zhang S-L, Xu T-Y, Yang Z-L, Han S, Zhao Q, Miao C-Y. 2018. Crystal structure-based comparison of two NAMPT inhibitors. Acta Pharmacol Sin 39:294–301. doi:10.1038/aps.2017.80 28858298 PMC5800471

[55] Li Y, Wang Z, Wang J, Jiang Z, Chen M, Ai H, Ma C, Tong Q, Liu L, Velkov T, Sun H, Pu J, Liu J, Dai C, Sun Y. 2025. ARRDC4-mediated glycolysis enhances innate immunity to influenza A virus through fructose-1,6-bisphosphate. Proc Natl Acad Sci USA 122:e2512385122. doi:10.1073/pnas.2512385122 40875808 PMC12415272

[56] Ke W, Zhou Y, Lai Y, Long S, Fang L, Xiao S. 2022. Porcine reproductive and respiratory syndrome virus nsp4 positively regulates cellular cholesterol to inhibit type I interferon production. Redox Biol 49:102207. doi:10.1016/j.redox.2021.102207 34911669 PMC8758914

[57] Liu P, Tang N, Meng C, Yin Y, Qiu X, Tan L, Sun Y, Song C, Liu W, Liao Y, Lin S-H, Ding C. 2022. SLC1A3 facilitates Newcastle disease virus replication by regulating glutamine catabolism. Virulence 13:1407–1422. doi:10.1080/21505594.2022.2112821 35993169 PMC9415643

[58] Blázquez A-B, Mingo-Casas P, Quesada E, Priego EM, Pérez-Perez M-J, Martín-Acebes MA. 2025. Lipid-targeting antiviral strategies: Current state and future perspectives. Antiviral Res 236:106103. doi:10.1016/j.antiviral.2025.106103 39947433

[59] Ampomah PB, Lim LHK. 2020. Influenza A virus-induced apoptosis and virus propagation. Apoptosis 25:1–11. doi:10.1007/s10495-019-01575-3 31667646

[60] Mehrbod P, Ande SR, Alizadeh J, Rahimizadeh S, Shariati A, Malek H, Hashemi M, Glover KKM, Sher AA, Coombs KM, Ghavami S. 2019. The roles of apoptosis, autophagy and unfolded protein response in arbovirus, influenza virus, and HIV infections. Virulence 10:376–413. doi:10.1080/21505594.2019.1605803 30966844 PMC6527025

[61] Zhou Q, Shi D, Tang Y-D, Zhang L, Hu B, Zheng C, Huang L, Weng C. 2024. Pseudorabies virus gM and its homologous proteins in herpesviruses induce mitochondria-related apoptosis involved in viral pathogenicity. PLoS Pathog 20:e1012146. doi:10.1371/journal.ppat.1012146 38669242 PMC11051632

