# Supplementary material for: The nicotinamide phosphoribosyltransferase inhibitor FK866 restricts influenza A virus replication by perturbing viral polymerase activity
Source: J Virol. 2026 Jun 15;100(7):e00591-26. doi: 10.1128/jvi.00591-26 (PMC13386833; doi:10.1128/jvi.00591-26)
Supplement: Supplemental figures — Fig. S1 to S5. [file jvi.00591-26-s0001.pdf]

The nicotinamide phosphoribosyltransferase inhibitor FK866 restricts influenza A virus replication by perturbing viral polymerase activity

Changjie Lv[1,2,3,4,#], Shuang Wang[1,2,4,#], Mingyue Song[1], Jianmei Wu[2], Wanxin Wei[1,2], and Guijie Guo[1,2,3,4,*]

[1] Key Laboratory of Animal Pathogen Infection and Immunology of Fujian Province, College of Animal Sciences, Fujian Agriculture and Forestry University, Fuzhou 350002, China

[2] Joint Laboratory of Animal Pathogen Prevention and Control of Fujian-Nepal, College of Animal Sciences, Fujian Agriculture and Forestry University, Fuzhou 350002, China

[3] Key Laboratory of Fujian-Taiwan Animal Pathogen Biology, College of Animal Sciences, Fujian Agriculture and Forestry University, Fuzhou 350002, China

[4] Engineering Research Center for Animal Breeding and Sustainable Production, College of Animal Sciences, Fujian Agriculture and Forestry University, Fuzhou 350002, China

[#]These authors contributed equally.

*Correspondence:

Guijie Guo, guoguijie@fafu.edu.cn, or guojie1125@163.com

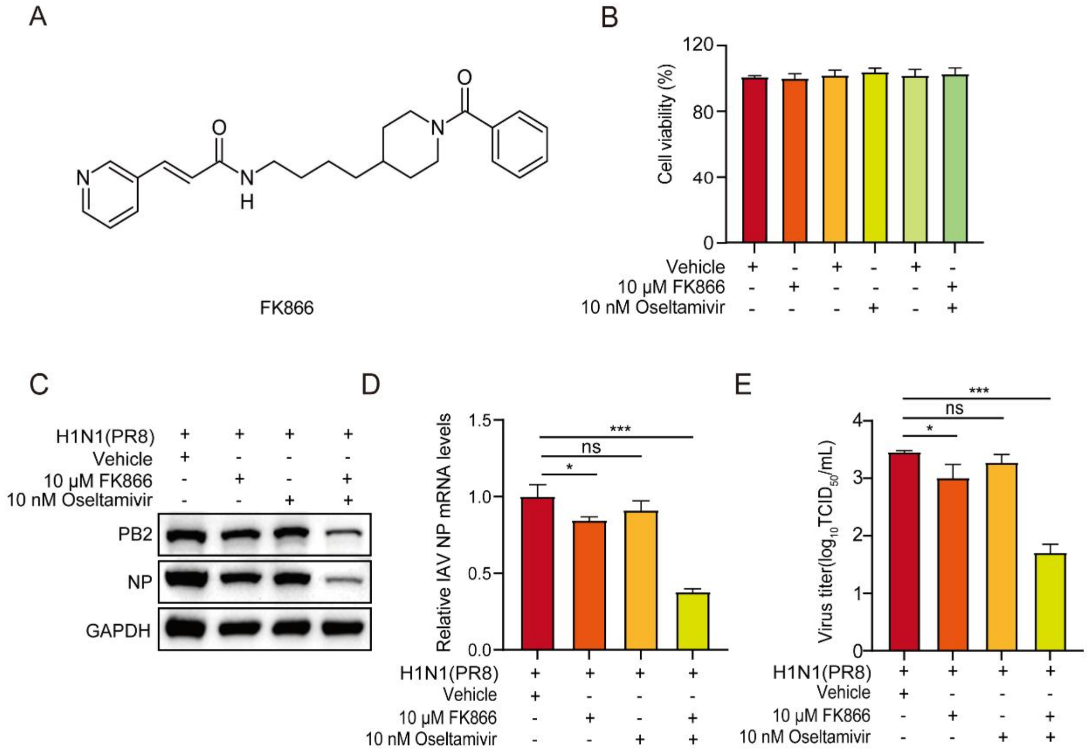

Figure S1. FK866 enhances the anti-IAV efficacy of oseltamivir

(A) Molecular structure formula of FK866. (B) The cytotoxicity of 10 μM FK866, 10 nM oseltamivir, and their combination (10 μM FK866 + 10 nM oseltamivir) on A549 cells was assessed after 24 h treatment. (C-E) Western blotting was performed to detect NP and PB2 proteins of H1N1 (PR8) in cells treated with 10 μM FK866, 10 nM oseltamivir, a combination of FK866 and oseltamivir (10 μM FK866 + 10 nM oseltamivir), or vehicle at 24 hpi (C). NP mRNA levels and viral titers were tested by qPCR and $TCID_{50}$ assays (D-E). Data are presented as mean ± SD of three independent experiments. **P* < 0.05, ****P* < 0.001, ns: non-significant.

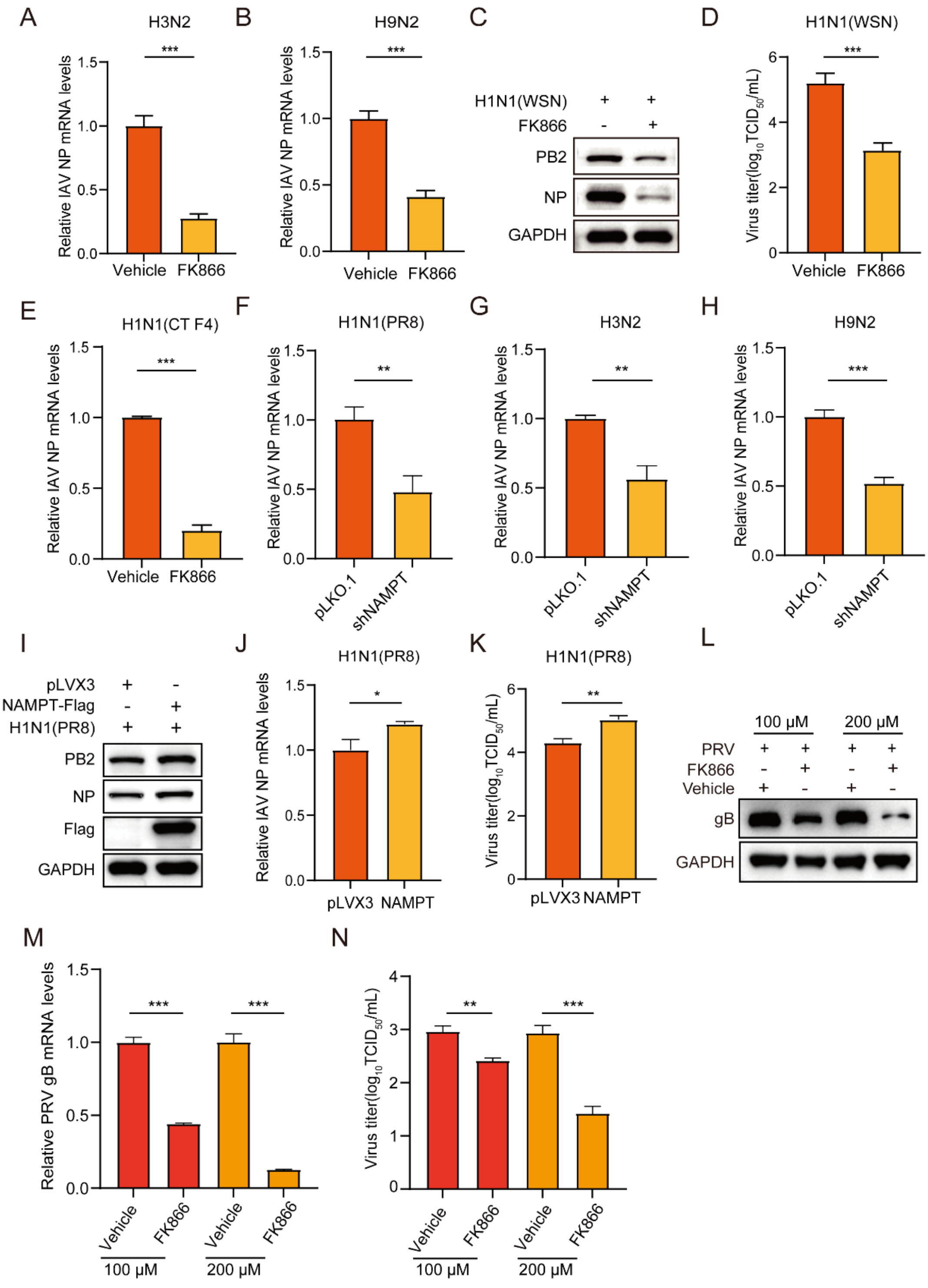

Figure S2. FK866 or depletion of NAMPT suppresses the replication of various IAV subtypes and PRV

(A-B) NP mRNA levels in FK866- and vehicle-treated A549 cells infected with H3N2 (A), or H9N2 (B) at an MOI of 0.01 for 24 h, were detected by qPCR. (C-D) NP and

PB2 protein levels, and viral titers in FK866- and vehicle-treated A549 cells infected with H1N1 (WSN) at an MOI of 0.01 for 24 h, were examined by Western blotting (C) and $TCID_{50}$ assays (D). (E) NP mRNA levels in FK866- and vehicle-treated A549 cells infected with H1N1 (CT F4) at an MOI of 0.01 for 24 h, were detected by qPCR. (F-H) Control and NAMPT knockdown A549 cells were infected with H1N1 (PR8) (MOI=0.01, 24 h) (F), H3N2 (MOI=0.01, 24 h) (G), or H9N2 (MOI=0.01, 24 h) (H). NP mRNA levels were examined by qPCR. (I-K) Control and NAMPT overexpressing A549 cells were infected with H1N1 (PR8) (MOI=0.01, 24 h). NP and PB2 protein levels, NP mRNA levels, and virus titers were measured by Western blotting (I), qPCR (J) and $TCID_{50}$ assays (K). (L-N) Western blotting was performed to detect PRV gB protein levels in FK866- and vehicle-treated cells infected with PRV for 24 h (L). The PRV gB mRNA levels were detected by qPCR (M). The viral titers were tested by $TCID_{50}$ assays (N). Data are presented as mean ± SD of three independent experiments. $*P < 0.05$, $**P < 0.01$, $***P < 0.001$.

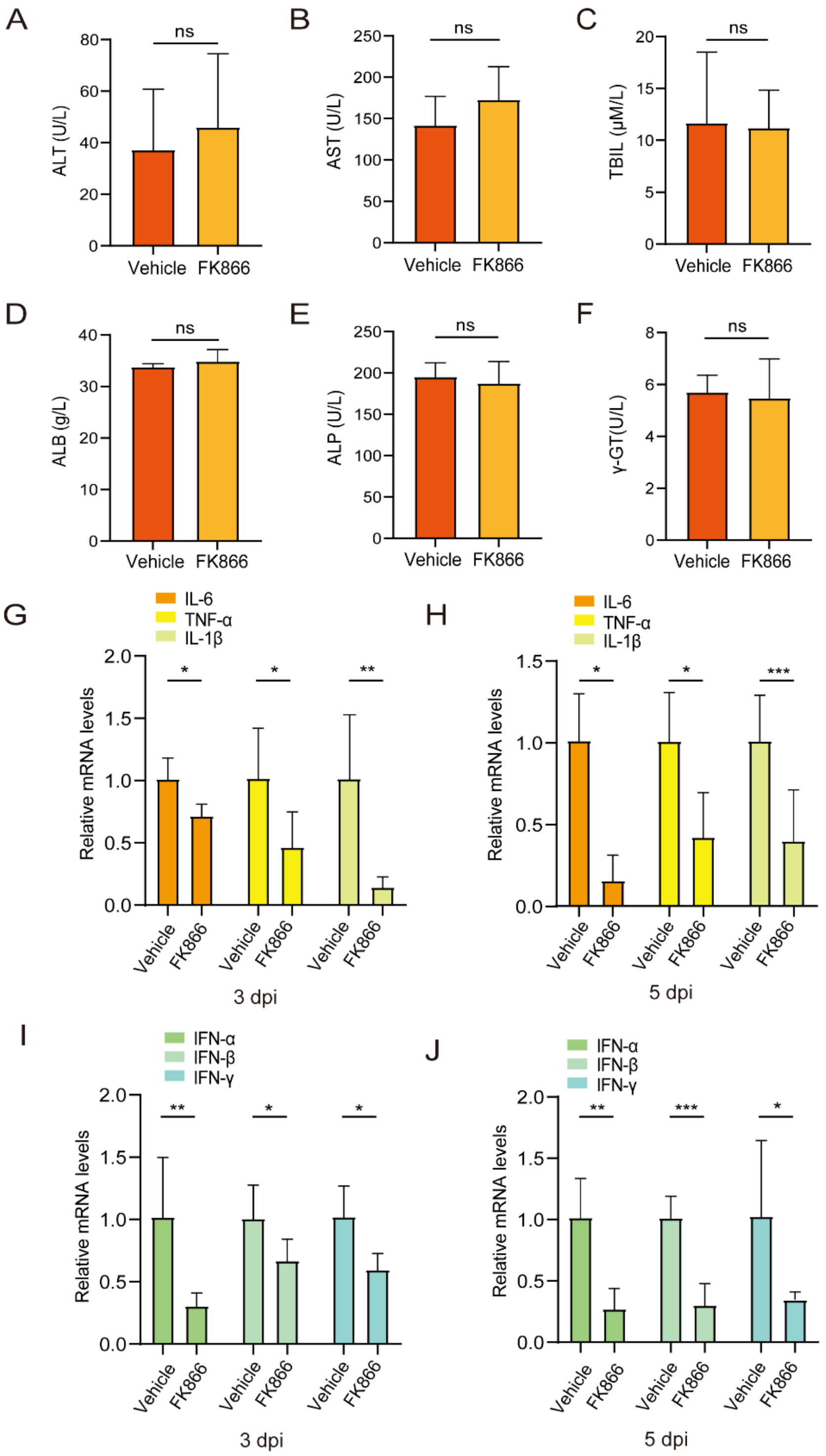

Figure S3. Hepatotoxicity and cytokine levels were assessed in mice

The mice received oral administration of 10 mg/kg FK866 or vehicle every other day for 21 days. (A-F) Serum biochemical markers including ALT (A), AST (B), TBIL (C), ALB (D), ALP (E), and γ-GT (F) were measured after 21 days. (G-H) The mRNA levels

of IL-6, TNF-α, and IL-1β were measured by qPCR in the lungs of vehicle- and FK866 (10 mg/kg)-treated mice infected with PR8 virus at 3 and 5 dpi. (I-J) The mRNA levels of IFN-α, IFN-β, and IFN-γ were measured by qPCR in the lungs of vehicle- and FK866 (10 mg/kg)-treated mice infected with PR8 virus at 3 and 5 dpi. Data are presented as mean ± SD. *$P < 0.05$, **$P < 0.01$, ***$P < 0.001$, ns: non-significant.

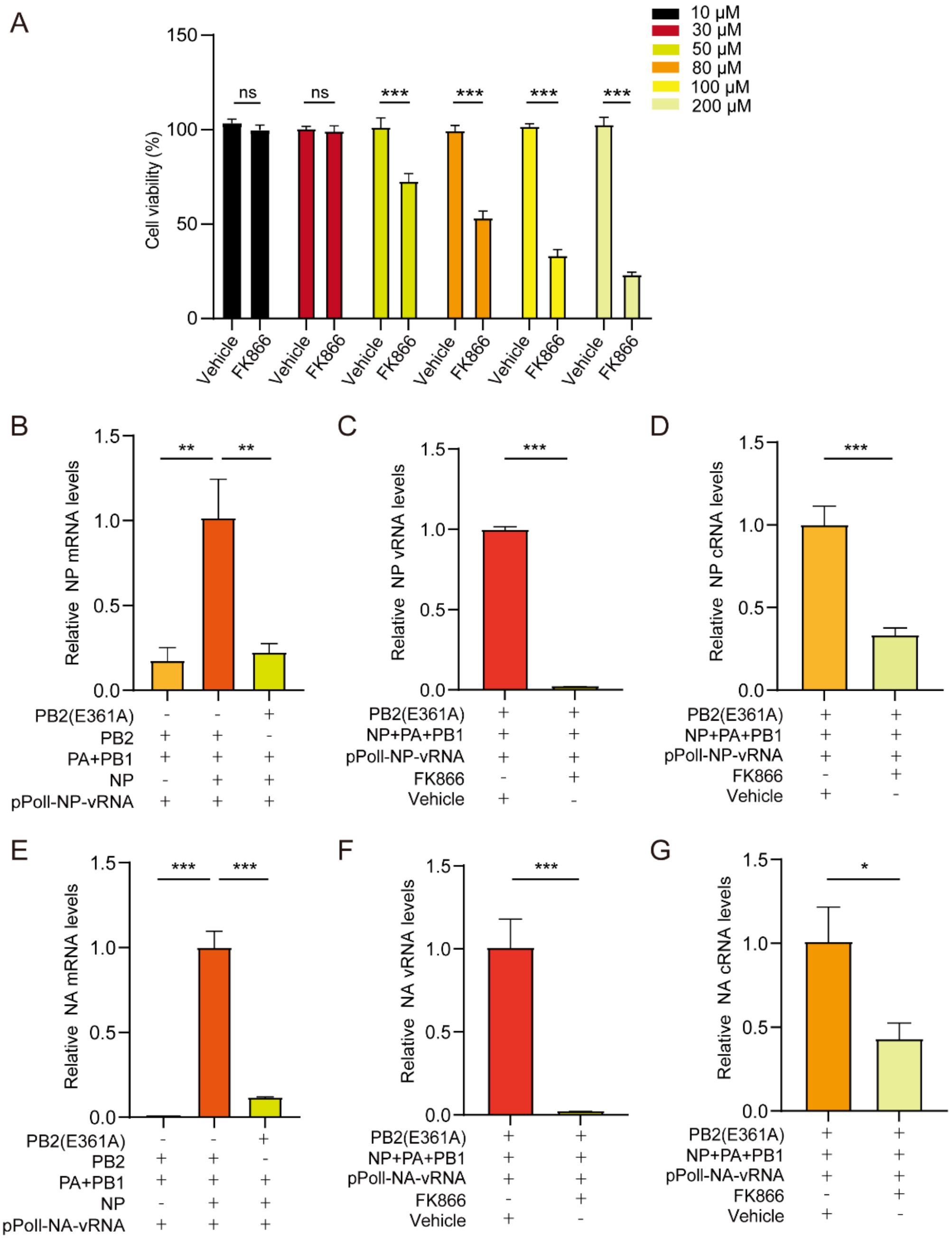

Figure S4. FK866 represses IAV transcription and replication

(A) The cytotoxicity of FK866 in HEK293T cells was assessed after 24 h of treatment at different concentrations. (B-D) HEK293T cells were transfected with plasmids encoding PB2 (E361A) or wild-type (WT) PB2, along with PB1, PA, NP, and pPol I-NP-vRNA. The NP mRNA, vRNA, and cRNA levels were detected by qPCR in the FK866- and vehicle-treated cells at 24 h post-transfection. (E-G) HEK293T cells were transfected with plasmids encoding PB2 (E361A) or WT PB2, along with PB1, PA, NP, and pPol I-NA-vRNA. The NA mRNA, vRNA, and cRNA levels were detected by qPCR in the FK866- and vehicle-treated cells at 24 h post-transfection. Data are presented as mean ± SD of three independent experiments. $^{*}P < 0.05$, $^{**}P < 0.01$, $^{***}P < 0.001$, ns: non-significant.

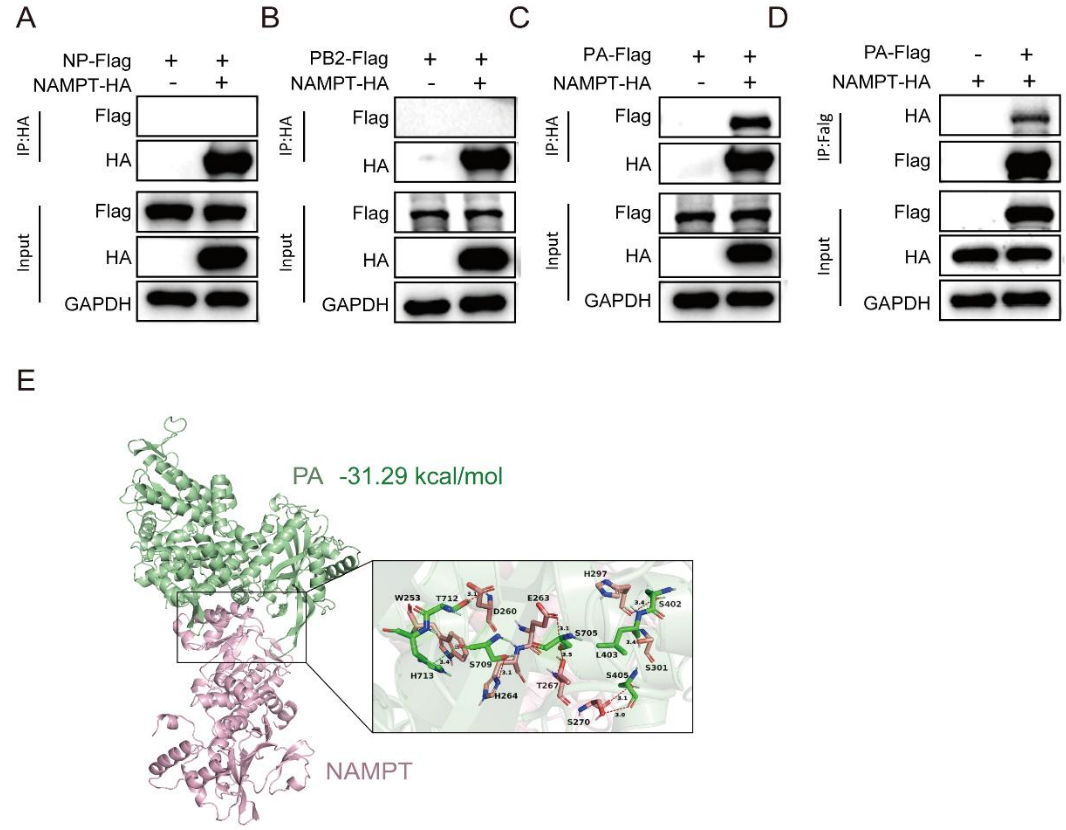

Figure S5. NAMPT interacts with viral PA protein

(A) HEK293T cells were transfected with NAMPT-HA and NP-Flag plasmids, followed by IP assays. NAMPT-HA was used as the bait protein to pull down NP-Flag. (B) HEK293T cells were transfected with NAMPT-HA and PB2-Flag plasmids, and subjected to IP assays. NAMPT-HA was used as the bait protein to pull down PB2-Flag.

(C) HEK293T cells were transfected with NAMPT-HA and PA-Flag plasmids, followed by IP assays. NAMPT-HA was used as the bait protein to pull down PA-Flag. (D) HEK293T cells were transfected with NAMPT-HA and PA-Flag plasmids, and PA-Flag was used as the bait protein to pull down NAMPT-HA by IP assays. (E) Molecular docking of NAMPT with PA was performed.
